# Transcriptome Sequencing of the Blind Subterranean Mole Rat, *Spalax galili*: Utility and Potential for the Discovery of Novel Evolutionary Patterns

**DOI:** 10.1371/journal.pone.0021227

**Published:** 2011-08-12

**Authors:** Assaf Malik, Abraham Korol, Sariel Hübner, Alvaro G. Hernandez, Jyothi Thimmapuram, Shahjahan Ali, Fabian Glaser, Arnon Paz, Aaron Avivi, Mark Band

**Affiliations:** 1 Institute of Evolution, University of Haifa, Haifa, Israel; 2 W. M. Keck Center for Comparative and Functional Genomics, University of Illinois, Urbana, Illinois, United States of America; 3 Bioinformatics Knowledge Unit, The Lorry I. Lokey Interdisciplinary Center for Life Sciences and Engineering, Technion - Israel Institute of Technology, Haifa, Israel; 4 Department of Human Molecular Genetics and Biochemistry, Sackler School of Medicine, Tel Aviv University, Tel Aviv, Israel; Virginia Tech, United States of America

## Abstract

The blind subterranean mole rat (*Spalax ehrenbergi* superspecies) is a model animal for survival under extreme environments due to its ability to live in underground habitats under severe hypoxic stress and darkness. Here we report the transcriptome sequencing of *Spalax galili*, a chromosomal type of S. *ehrenbergi*. cDNA pools from muscle and brain tissues isolated from animals exposed to hypoxic and normoxic conditions were sequenced using Sanger, GS FLX, and GS FLX Titanium technologies. Assembly of the sequences yielded over 51,000 isotigs with homology to ∼12,000 mouse, rat or human genes. Based on these results, it was possible to detect large numbers of splice variants, SNPs, and novel transcribed regions. In addition, multiple differential expression patterns were detected between tissues and treatments. The results presented here will serve as a valuable resource for future studies aimed at identifying genes and gene regions evolved during the adaptive radiation associated with underground life of the blind mole rat.

## Introduction

The blind mole rat *Spalax ehrenbergi* species complex (*Muroidea* superfamily, *Spalacidae* family, *Spalax* genus) is a group of subterranean rodents, prevalent throughout the eastern Mediterranean region, ranging from southern Turkey through North Africa. Various chromosomal types of *Spalax* were identified, with diploid numbers ranging from 2n = 52 to 2n = 62. *Spalax* lives in a complex underground tunnel system, inhabited by single individuals and characterized by fluctuating oxygen levels and darkness. In field measurements during the rainy season, oxygen levels in the burrows were detected at 7% with CO_2_ levels around 6% [1]. In the laboratory, *Spalax* survives at 3% O_2_ for up to 11 hours, as compared to only 2–4 hours for rats [2]. Structural and functional changes in the skeletal muscles, cardiovascular and respiratory systems were suggested to play a key role in *Spalax* evolution. Compared with other rodent species *Spalax* has a higher density of blood vessels in muscle tissues, increased lung diffusion capacity and a higher erythrocyte count [3], [4]. Similarly, *Spalax* heart rate is about 40% of the expected rates for animals of similar size, reflecting increased aerobic-capacity especially during tunnel-system construction under hypoxic conditions [5], [6].

In recent years, studies have started to unravel the molecular pathways and mechanisms involved in response to hypoxia in *Spalax*. Differential expression patterns among many genes have been identified between *Spalax* chromosomal types, and between *Spalax* and *Rattus*. These genes include *hemoglobin*, *myoglobin*, *haptoglobin*, *neuroglobin* and *cytoglobin*, among others [7]–[12]. Likewise, *hypoxia inducible factor1-alpha*, *erythropoietin (Epo)*, and *Epo* receptors were found to exhibit different expression patterns in *Spalax* as compared to *Rattus* throughout development [13], [14]. Furthermore, patterns of expression in many tumor types resemble those found normally in *Spalax*. For example, in both cases there is an over-expression of *p53* associated genes that enable the cell to escape hypoxia induced apoptosis by favoring reversible cell cycle arrest [15]–[17]. Accordingly, the binding domain of *Spalax p53* harbors two amino acid substitutions identical to those found in *p53* expressed in human tumor cells, resulting in increased activation of DNA repair elements and reduced activation of apoptotic genes [15]. Similarly, *vascular endothelial growth factor (VEGF)*, a major neoangiogenic factor, is constitutively expressed at high levels in *Spalax* muscles with no increased transcription under hypoxia [18], [19], analogous to cancers [20], [21] and contradictory to the response reported in other mammals and in normal cells. These results suggest that *Spalax* may have a significant potential as a model in cancer research.

*S. ehrenbergi* has undergone, what was described as “peculiar” central nervous system evolution [22]. The size of *Spalax* brain and skull was found to be significantly larger than that of species of comparable dimensions, presumably reflecting enlargement of motor centers specializing in tunnel-digging [22], [23]. Additional changes in the brain include progressions of somatosensory regions involved in olfactory/auditory/tactile perception required for movement in darkness accompanied by regressions in ocular centers [24], [25]. Different changes in morphology (e.g., absence of tail and neck, broad flat head, and reduced external ear) reflect adaptations to movement inside the tunnel structure. Similarly, the morphological evolution of *Spalax* claws, incisors and muscle system allowed adaptation to digging behavior [26]. An additional interesting morphology is the eye of *Spalax*, which is atrophied, subcutaneous and is not used for visual identification of objects, however, allows a circadian rhythm behaviorally and molecularly [27]–[30].

Here we present the results of the first large-scale transciptome sequencing project conducted from S. *ehrenbergi* cDNA libraries derived from hypoxic and normoxic muscle and brain libraries using Sanger, GS FLX and GS Titanium pyrosequencing technologies. cDNAs of the chromosomal type *S. galili* (2n = 52) were sequenced and assembled. Over 51,000 isotigs were mapped to more than 11,000 predicted non-redundant genes expressed in brain and muscle tissues under hypoxia and normoxia. Based on these results, we identified and analyzed non-conserved transcribed regions unique to *S. galili*, large numbers of alternatively spliced variants, large non-conserved open reading frames (ORFs) and short nucleotide polymorphisms (SNPs). In addition, genes exhibiting significant over-expression in different tissues and treatments were identified based on annotated read counts. Finally, we investigated a possible functional protein change as the result of a novel coding insert through structural modeling.

## Methods

### Ethics statement

All animal handling protocols were approved by the Haifa University Committee for Ethics on Animal Subject Research, permit # 193/10 and approved by the Israel Ministry of Health. Permit # 193/10 covers all protocols and experimentation involving *Spalax*, rats or mice used in this experiment. This is a renewable permit which is current from July 2010– July 2014. The permit covers the number of animal subjects, housing conditions, veterinary regulations and inspections, hypoxia treatments and sacrifice methods for this experiment. No permits for capturing *Spalax* in unprotected areas are required (Israel Nature Reserves Authority; letter provided to editorial office).

### Animals

*S. galili* individuals were captured in the field near the village of Kerem Ben Zimra in northern Israel and housed under ambient conditions in individual cages. At least three individuals were used for each experiment. *S. galili* individuals were placed in a 70×70×50 cm chamber divided into separate cells, and the chosen gas mixture was delivered at 3.5 l/min. Animals were exposed to three hypoxic conditions: (1) 6% O_2_, i.e. the lowest oxygen levels recorded in S. *ehrenbergi* tunnels after rainstorms [1]; (2) 10% O_2_, for up to 44 hours, i.e. the estimated time/oxygen-levels during tunnel reconstruction; (3) 3% O_2_, i.e. critical oxygen levels for survival in S. *ehrenbergi* [2]. For the forgoing conditions, different exposure-times were chosen for different experiments.

### Tissues and total RNA

Animals were sacrificed by injection with Ketaset CIII (Fort Dodge, USA) at 5 mg/kg of body weight. Brain and skeletal muscle were removed and immediately frozen in liquid nitrogen. Total RNA was extracted using TRI Reagent (Molecular Research Center, Inc.) following the manufacturer's instructions.

### Sanger sequencing

Total RNAs were extracted from *Spalax* brain and muscle from animals exposed to different levels of oxygen: brain normoxic, brain 3% 8 hours, brain 6% 5 hours, brain 6% 10 hours, brain 10% 24 hours, brain 10% 44 hours; muscle normoxic, muscle 3% 8 h, muscle 6% 5 h, muscle 6% 10 h, muscle 10% 24 h, muscle 10% 44 h. Samples were combined into separate brain or muscle pools.

Poly(A)+mRNA was isolated from total RNA using the Oligotex Direct mRNA kit (Qiagen). Two micrograms of poly(A)+mRNA were converted to double stranded cDNA using the Creator Smart cDNA library construction kit (Clontech). For the primary library, an aliquot of the cDNA was digested with SfiI, size selected (>500 bp) and cloned into the pDNR-LIB plasmid vector (Clontech). Double stranded cDNA was normalized using the Trimmer-direct kit (Evrogen). Double stranded cDNA was denatured at 980C for 2 minutes in hybridization buffer (50 mM Hepes, pH = 7.5 and 0.5 M NaCl) and allowed to renature for 5 hours at 68°C. Double stranded cDNAs (i.e. abundant transcripts) were cleaved using Duplex-Specific Nuclease. Complementary cDNAs that remained single stranded were amplified by PCR as described in the manufacturer's protocol, digested with SfiI and size selected (>500 bp). Normalized cDNAs were cloned into pDNR-LIB (Clontech). The libraries were transformed using DH10B electrocompetent cells. Transformed colonies were picked and plated in 384 well plates in LB containing ampicillin. Plasmids were extracted and sequenced on Applied Biosciences 3730 sequencers. Base calling with quality score was carried out using Phred. Sequences were considered high quality with average Phred scores of 20 or above and a minimum of 200 bp. Vector sequence was detected and trimmed using Cross-Match. In total, 7663 *Spalax* high quality Sanger sequences were produced.

### 454 GS FLX sequencing

A normalized cDNA library was created using a pool of RNA extracted from muscle or brain of individual mole rats following exposure to normoxic (21% O_2_), acute short term hypoxia (3%, 5 hours) or moderate long term hypoxia (10% 44 hours). The library was created as previously published [31].

### RNA isolation, cDNA synthesis and normalization for GS FLX

mRNAs (PolyA RNA) were isolated from pooled mole rat brain and muscle RNA with the Oligotex Mini Kit (Qiagen). cDNAs were synthesized from 500 ng of mRNA following the Clontech Creator SMART cDNA synthesis system using modified Oligo-dT (for compatibility with GS FLX) and 5′ RACE primers. The primers sequences are: CDSIII-First 454: 5′ TAG AGA CCG AGG CGG CCG ACA TGT TTT GTT TTT TTT TCT TTT TTT TTT VN 3′ and SMARTIV: 5′ AAG CAG TGG TAT CAA CGC AGA GTG GCC ATT ACG GCC GGG 3′.

For normalization, 300 ng of cDNA were denatured and allowed to self anneal in a 1× hybridization buffer (50 mM Hepes, pH 7.5 and 0.5 M NaCl) for a period of 4 hrs. After hybridization, DSN (Duplex/double stranded specific Nuclease; Evrogen, Russia) was added to the reaction to degrade ds-cDNAs. Single stranded transcripts were PCR amplified to create normalized ds-cDNAs.

### Library preparation (DNA processing) for GS FLX

cDNAs were nebulized and size selected for an average size of 400–500 bp. GS FLX specific adapters, Adapter A and Adapter B, were ligated to the cDNA ends after an end polishing reaction. The adapter ligated DNAs were then mobilized to the library preparation beads and single stranded template (sst) cDNAs were captured.

### Emulsion PCR, enrichment and DNA bead loading

Emulsion PCR (emPCR) reactions were set up for titration runs using 6×105, 2.4×106, 4.8×106 and 9.6×106 molecules of sstcDNAs corresponding to 0.5, 2, 4 and 8 copies of the sstcDNA per bead. Following the titration run a full 70×75 PicoTiterPlate (PTP) was run on the Roche 454 sequencer.

FLX sequencing runs produced a total of 433621 sequences with an average read length of 200 bases for a total of over 86 megabase of expressed sequence.

### GS FLX Titanium sequencing

A tagged normalized library was constructed from 4 tissue sources, brain normoxic (21%, O_2_); brain hypoxic, (3% O_2_); muscle normoxic and muscle hypoxic. Messenger RNA was isolated from total RNA using the Oligotex mRNA Mini kit (Qiagen, CA). First and second strand cDNA were synthesized from 200 ng of mRNA using the SuperScript® Double-Stranded cDNA Synthesis Kit (Invitrogen, CA) with 100 µM random hexamer primers (Fermentas, USA). Double-stranded cDNA was cleaned up with a QIAquick Minelute PCR purification column (Qiagen, CA) and nebulized with the nebulization kit supplied with the GS Titanium Library Preparation kit (454 Life Sciences, Branford, CT) following the manufacturer's recommendations (30 psi for 1 minute). Sheared cDNA was cleaned with a QIAquick PCR minelute column and blunt-ended. A dA-overhang was added at the 3′ end using Klenow exo-minus polymerase (5 U/µl) (NEB). Titanium adaptors (454 Life Sciences, Branford, CT) were added by adding 9 µl water, 25 µl 2× Rapid Ligase buffer (Enzymatics, MA) 5 µl (50 µM) Titanium adapter A/B mix and 1 µl T4 DNA Ligase (600 U/µl (Enzymatics, MA) and incubated at room temperature for 15 minutes. Adaptor ligated cDNA was run on an E-GEL EX 2% agarose gel (Invitrogen, CA) following the manufacturer's instructions and cDNAs in the size range of 400–800 bp were excised from the gel and purified with a Qiagen Gel Extraction kit. One µl of the gel- purified cDNA was used as template for amplification in a 50 µl PCR reactions containing 10 µl 5× Phusion Buffer HF (NEB), 25 µM Adapter A primer (5′CCATCTCATCCCTGCGTGTCTCCGACTCAG ACGAGTGCGT3′), 25 µM Adapter B primer (5′CCTATCCCCTGTGTGCCTTGGC AGTCTCAGT3′), 3% DMSO, 10 mM dNTPs and 1 U Phusion polymerase (Finnzymes/NEB, USA). The PCR conditions were as follows: 98°C for 30 seconds, followed by 15 cycles with 98°C for 10 seconds, 68°C for 30 seconds and 72°C for 30 seconds, with a final extension of 72°C for 5 min.

### Normalization of cDNA library

The cDNA library was normalized with the Trimmer Direct Kit (Evrogen, Russia). In brief, 300 ng of cDNA were incubated at 95°C for 5 minutes followed by incubation at 68°C for 4 hours in the hybridization buffer included in the kit (50 mM Hepes, pH 7.5 and 0.5 M NaCl). After the incubation, the reaction was treated with ¼ units of duplex specific nuclease (DSN). The normalized cDNA was then amplified from 1 µl of DSN-treated cDNA by PCR using primers complementary to adaptors A and B with the following conditions: 98°C for 30 seconds, followed by 10 cycles with 98°C for 10 seconds, 68°C for 30 seconds and 72°C for 30 seconds, with a final extension of 72°C for 5 minutes.

### GS FLX Titanium sequencing procedures

Following library construction, the samples were quantified using a Qubit fluorometer (Invitrogen, CA) and average fragment sizes were determined by analyzing 1 µl of the samples on the Bioanalyzer (Agilent, CA) using a DNA 7500 chip. The 4 barcoded libraries were mixed in equimolar concentrations. The pooled library was diluted to 1×106molecules/µl. Emulsion-based clonal amplification and sequencing on the GS FLX Titanium system were performed in the W. M. Keck Center for Comparative and Functional Genomics at the University of Illinois at Urbana-Champaign. Signal processing and base calling were performed using the bundled 454 Data Analysis Software.

### Assembly

The adaptors used for cDNA library construction were trimmed using cross-match (www.phrap.org) and GS FLX reads for each tissue were sorted based on the barcodes before de novo assembly. For GS FLX Titanium Sequencing, the reads of specific tissue/treatment were assembled separately using Newbler version 2.3 (454 Life Sciences). In addition, a combined assembly of all Titanium, FLX and Sanger sequences was created. Newbler constructs ‘isotigs’ based on different combinations of contigs (exons) belonging to the same contig-graph, which are grouped in ‘isogroups’. The distinction between isotigs and isogroups is similar to that between transcripts and genes, respectively, yet a homology-based analysis was needed in order to biologically validate the results.

For each isotig, read identities, sequences, alignments, and the consensus sequence, were retrieved from Newbler ACE files, using Biopython tool. Based on the components of the isotigs, it was possible to detect candidate single nucleotide polymorphism alleles, to distinguish between different groups of reads (e.g., reads produced in different sequencing runs), and to check the quality/depth and coverage of isotigs. In addition, Newbler ACE files analysis procedures were tested, by visually inspecting the validity of the results in multiple isotigs, using Tablet and Consed assembly viewers [32], [33]. All Sanger sequences have been submitted to GenBank (accession numbers JG745372–JG753034), .SFF files have been submitted to the GenBank Short Read Archive (SRA) accession number SRA031271.1. Assembled isotigs were deposited in the Transcriptome Shotgun Assembly (TSA) database, accession numbers JL968997–JL999999 and JO000001–JO020426 . BLAST results against the Swissprot and Mouse Refseq databases are presented in Table S1.

### Homology-based annotation

Using the Blastn program [34], isotig sequences were compared to the target Ensembl transcript databases of mouse, rat, cavia, human, rhesus, marmoset, dog, cow, and horse. Using the UCSC genome browser ensGene tables corresponding to the forgoing Ensembl transcripts it was possible to map predicted *S. galili* exons to genomic position in the reference species [35]. For each hit, a chaining procedure was conducted: all subsets of high-scoring segment pairs (HSPs), representing query/target syntenic regions, were first identified, and the subset with the largest coverage-size was selected. By ‘synteny’ we mean here: sequential order of hits along the query and the target sequences. The resulting chained-hits include isotig segments mapped to both transcripts and genomic-regions of mouse/rat/human. For each query, a single hit was classified as ‘unique’, if the total size of its HSPs, and their corresponding bit-scores, were at least 2 times larger than that of any other hit. The forgoing condition was irrelevant in hits mapped to the same target genomic regions, such as different transcripts belonging to the same gene.

Using the UCSC genome browser EnsGene tables, the identity of homologous exonic regions was predicted (i.e., coding-sequences, 5′/3′ un-translated regions, and non-coding genes). Names and properties of mouse/rat/human genes predicted to be orthologous were taken from the Ensembl Biomart database.

For selected isotigs (e.g. isotigs with non-conserved segments, based on Blast against transcripts), Blast search against mouse, rat, cavia, rabbit, and human genomes was performed. In addition Blast search against the entire NCBI nr/nt collection and reference genomic sequences, was performed for selected isotigs.

Detection of isotig homology to reference species genomic regions was conducted using Lastz (unpublished, Harris 2010), previously known as Blastz [36].

### Prediction of transcripts and proteins

For each isotig, a multiple-sequence alignment with the mapped reference transcripts was constructed using Mafft [37]. Based on the known open reading frames (ORFs) in the reference species, it was possible to translate the isotig sequence into predicted *S. galili* protein, or protein segment. This procedure also enabled the location of premature termination codons within the predicted transcripts, and identification of mutations in ORFs, and untranslated regions.

Phylogenetic tree, based on alignment of ORF regions, were built using the PhyML program [38]. Consensus tree was built using Consense in the Phylip package [39].

### Detection of novel transcribed regions

Based on similarity searches against human/mouse/rat genomes and transcriptomes, it was possible to detect *S. galili* novel exons and insertions in exons with no evidence for conservation beyond S. *ehrenbergi*. In order to exclude regions representing assembly errors or intron contamination we selected only those non-conserved regions flanked by conserved regions. It seems that a limited power of the similarity search programs to detect weak similarities between alternatively spliced *S. galili* sequences and target genomic regions may in some cases lead to false-negative and false-positive detection of similar sequences. In order to increase the detection power for novel regions we also locally aligned putative novel *S. galili* exons and their flanking regions against different mammalian introns mapped between the flanking regions, using the Mafft program. We used gap opening penalties higher than the Mafft default, in order to prevent fragmentation of exons into multiple alignment blocks. This procedure allowed us to filter out regions mistakenly identified as non-conserved based on Blast and Lastz results. On the other hand, insertions in exons can be reported to be non-conserved without implementing this procedure, as the absence of similar insertions in homologous sequences of several other species, may serve as an indication for lack of conservation.

### Comparative modeling of protein 3D structure

Predicted *S. galili* protein sequences were submitted to the HHpred server [40] in order to detect protein homology to known proteins. Models were constructed using MODELLER [41] based on the best templates available. Molecular graphics images were produced using the UCSF Chimera package [42]. Domains were colored based on the PFAM database [43].

### Identification of differentially expressed genes

In order to identify differentially expressed genes we used the Degseq program [44] which is implemented in R (www.R-project.org). Random sampling model of reads distribution (MARS) was selected, and P-values were calculated for each gene and adjusted to multiple testing by two alternative strategies [45], [46] in Degseq. In addition, expected and observed numbers of reads were compared using a *x* ^2^ test.

## Results

### Sequencing and assembly

Using *S. galili* (n = s52) cDNA pools, the following sequencing runs were conducted:

- Sanger sequencing for two individual normalized muscle and brain libraries derived from mixed treatments (Normoxic (21%), 3% 6 hrs., 6% 5 hrs., and 10%, for 22 hrs. and 44 hrs.).
- GS FLX sequencing for one combined normalized brain and muscle library using the same samples as above.
- GS FLX Titanium sequencing for the following four normalized libraries:
  (C.1) brain under normoxia (21% O_2_);
  (C.2) brain under hypoxia (3% 6 hrs. and 10% O_2_, for 44 hrs.);
  (C.3) muscle under normoxia (21% O_2_);
  (C.4) muscle under hypoxia (3% 6 hrs. and 10% O_2_, for 44 hrs.);

In total, cDNAs from six different sources were sequenced, to produce 7663 (Sanger), 846,747 (FLX), 281,098 (C.1), 484,928 (C.2), 466,452 (C.3), and 1,126,080 (C.4) Titanium reads, respectively. The average trimmed read length was 636, 218, 378, 373, 367, and 330 bp, respectively. Reads from all six sources were assembled together using Newbler 2.3 software to produce a total of 51,885 isotigs, with an average length of 1588 bp. In addition, isotigs originating from the same contig-graph were grouped into isogroups by Newbler, potentially reflecting multiple splice variants. Contigs that were not combined into isotigs may reflect genes that are not alternatively spliced, or small segments of cDNAs (for simplicity, these contigs are also referred to as isotigs here).

### Homology-based annotation of isotigs

**Figure 1:**
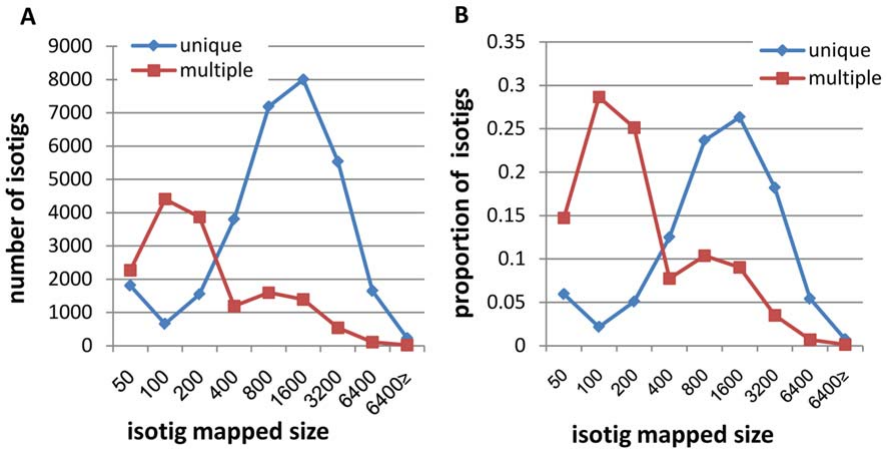
Count and proportion of annotated isotigs as a function of HSP size. This data refers to isotigs from the combined assembly, based on Blast results against mouse transcripts. Left: the total number isotigs with unique or multiple hits for different HSP sizes. Right: the proportion of isotigs with unique or multiple hits for different HSP sizes.

Among the total 51,885 isotigs from the combined assembly, 45,700, 43,600, and 44,500 isotigs showed significant homology to known mouse, rat, and human transcripts, respectively (see Methods). As multiple hits were generated for most isotigs, a procedure aimed to screen unique hits was employed (see Methods). Accordingly, ∼70% of the isotigs were uniquely mapped to mouse/rat/human genes (Figure S1). In addition, uniquely mapped isotigs exhibited higher similarity to their hits, and had longer HSP sizes (bp), as compared to those mapped to multiple genes (Figure 1).

In order to eliminate biases that can result from analyzing redundant isotigs representing possible variants of the same gene, we clustered all unique isotigs that mapped to the same reference genes. Accordingly, 12,107, 11,406, and 12,074 clusters were found, based on mouse, rat, and human genes, respectively (Figure S1). Isotigs with the largest HSP coverage were chosen as representative of each cluster. In addition, for each cluster, non-overlapping isotigs mapped to different regions of the same gene, were also selected. Thus a total of 18,022, 15,317, and 18,518 unique non-overlapping isotigs, representing genic regions were found, based on mouse, rat, and human genes, respectively (Figure S1).

**Figure 2:**
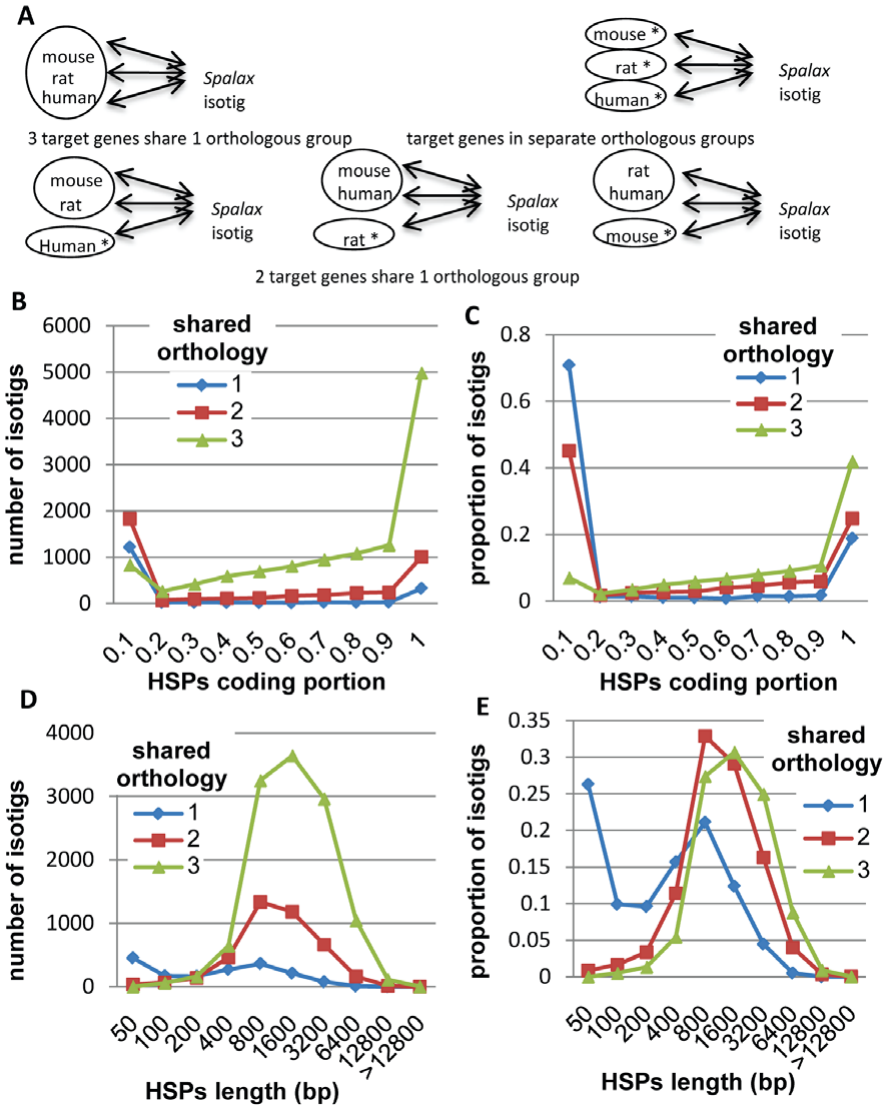
Shared orthology categories. (**a**) Category-3: Three target genes share one orthologous group (upper left). Category-2: Two target genes share one orthologous group (below). Category-1: the target genes are mapped to separate orthologous groups (upper right). Asterisk, near species name, indicates that the target gene is mapped to separate orthologous group, or that no hit was found. (**b**) The number of isotigs in each category (i.e., 1, 2, and 3) as a function of the HSP coding portion, measured as the total isotig size mapped to target coding region divided by the size mapped to both the coding and untranslated regions; (**c**) The proportion of isotigs in each category as a function of the HSP coding portion. (**d**) The number of isotigs in each category as a function of the total length mapped to target transcript. (**e**) The proportion of isotigs in each category as a function of the total length mapped to target transcript.

In order to test the consistency of annotations, mouse/rat/human target genes were classified in one of the following three categories: Category-3: the three target genes share one orthologous group; Category-2: two target genes share one orthologous group; Category-1: the target genes are mapped to separate orthologous groups (Figure 2a). The three categories (3, 2, and 1) included 11,884, 4,061 and 1,719 non-overlapping isotigs and were mapped to 8,774, 2,106 and 1,016 gene clusters respectively (Figure S1). Only 6% of all non-overlapping isotigs belonging to category 3 included HSPs mapped completely, or almost-completely, to un-translated regions of target transcripts, as compared to 45% and 71% for categories 2, and 1, respectively (Figure 2b, c). Similarly, the isotigs in category 3 had a median aligned size distribution of 800–1600 bp, as compared to 400–800 bp for category 2, and <400 bp for category 1 (Figure 2d, e). Therefore, the results for many of the isotigs falling into classifications 1 and 2 can be partly attributed to similarity with weakly conserved regions such as 3′ UTRs.

### Estimated transcription levels

For each non-redundant isotig, the number of reads belonging to sources C.1, C.2, C.3, and C.4 were counted (i.e., n1, n2, n3, and n4), and expected counts were calculated based on the total counts of reads for the corresponding sources (i.e., N1, N2, N3, and N4). Using Gene Ontology (GO) analysis, we tested whether differences in relative frequencies of reads reflect differential expression of genes characteristic of specific tissues/treatments (Figure S2). Genes estimated to be significantly enriched (P values<5×10^−2^, see Methods) in *S. galili* brain (C.1 vs. C.3, and C.2 vs. C.4), and muscle (C.3 vs. C.1, and C.4 vs. C.2), were compared to background groups consisting of all genes found in each comparison. The results indicate highly significant (after FDR correction) over-representation of brain and muscle related terms in the groups of genes estimated to be enriched in *S. galili* brain and muscle tissues, respectively (Tables 1 and 2). On the other hand, for genes estimated to be up-regulated under hypoxia (C.1 vs. C.2, and C.3 vs. C.4), only few terms remained significant after FDR correction. Terms that remained significant after FDR correction in C.4 vs. C.3 (muscle libraries) include blood vessel development, vasculature development, regulation of catabolic processes, regulation of cell proliferation, proteinaceous extracellular matrix, and additional terms related to extracellular regions (Table 3). Most of these terms represent processes known to be up-regulated in hypoxia. Details for the top 50 *Spalax* genes which exhibit read count differences of the highest significance are shown in Tables S2 and S3. Note that many of these genes were previously identified in *Spalax* microarray experiments [47], further supporting these results. As would be expected a much larger number of genes show enrichment between tissues as opposed to the comparison between oxygen levels within tissues. Normalization of the libraries generally reduces the abundance of very high frequency transcripts and enhances discovery of rare transcripts, however, despite this process we were able to recognize tissue and hypoxia signatures among the 4 libraries.

**Table 1:** Top GO terms enriched in 2840 *Spalax* genes significantly (*P* value<0.05) up-regulated in brain/normoxia vs. muscle/normoxia.

Term	Count	*P* value	FDR
synapse	178	1.2E-31	6.8E-29
neuron projection	188	5.9E-30	1.7E-27
synapse part	127	1.9E-25	3.6E-23
cell projection	243	1.9E-23	2.8E-21
plasma membrane	525	3.0E-23	3.5E-21
axon	97	4.9E-19	4.7E-17
neurological system process	185	1.9E-20	8.0E-17
transmission of nerve impulse	117	6.8E-18	1.4E-14
synaptic transmission	98	1.1E-17	1.6E-14
synaptic vesicle	52	3.4E-16	2.8E-14
regulation of transmission of nerve impulse	74	1.7E-16	2.3E-13
plasma membrane part	320	4.6E-15	3.3E-13
cell junction	144	1.1E-14	6.8E-13
dendrite	98	1.6E-14	9.1E-13
cell-cell signaling	113	1.7E-15	1.4E-12
regulation of synaptic transmission	70	3.8E-15	2.6E-12
regulation of neurological system process	74	5.5E-15	3.2E-12
intrinsic to membrane	583	3.0E-13	1.6E-11
cytoplasmic vesicle	180	3.6E-13	1.7E-11
clathrin-coated vesicle	63	1.0E-12	4.7E-11
neuron development	114	1.0E-13	5.3E-11
vesicle	185	1.9E-12	7.9E-11
cytoplasmic membrane-bounded vesicle	158	3.0E-12	1.2E-10
membrane-bounded vesicle	162	5.0E-12	1.8E-10
synaptosome	55	1.3E-11	4.4E-10
neurotransmitter transport	52	1.2E-12	5.3E-10
cell soma	92	1.8E-11	5.5E-10
cell projection part	88	1.7E-11	5.5E-10
integral to membrane	550	4.0E-11	1.2E-09
coated vesicle	67	4.6E-11	1.3E-09
neuron projection development	95	4.0E-12	1.6E-09
synaptic vesicle membrane	23	1.6E-10	4.1E-09
neuron differentiation	130	1.5E-11	5.6E-09
ion transport	163	4.4E-11	1.5E-08
membrane fraction	189	6.2E-10	1.6E-08
gated channel activity	83	3.7E-11	5.1E-08
cytoskeleton	236	3.2E-09	7.7E-08
insoluble fraction	197	4.4E-09	1.0E-07
exocytosis	53	5.2E-10	1.6E-07
secretion by cell	72	6.0E-10	1.8E-07
site of polarized growth	36	4.5E-08	1.0E-06
growth cone	36	4.5E-08	1.0E-06
cation transport	124	3.9E-09	1.1E-06
behavior	111	4.6E-09	1.2E-06
cell projection organization	111	6.0E-09	1.5E-06
generation of a signal involved in cell-cell signaling	42	7.4E-09	1.5E-06
secretion	78	7.4E-09	1.6E-06
neurotransmitter secretion	32	7.0E-09	1.6E-06
ion channel activity	91	2.6E-09	1.8E-06
substrate specific channel activity	92	6.9E-09	2.4E-06
channel activity	94	5.5E-09	2.5E-06
passive transmembrane transporter activity	94	5.5E-09	2.5E-06
clathrin coated vesicle membrane	28	1.2E-07	2.6E-06
monovalent inorganic cation transport	82	1.3E-08	2.6E-06
cell projection morphogenesis	78	1.4E-08	2.7E-06
voltage-gated ion channel activity	59	1.1E-08	3.0E-06
voltage-gated channel activity	59	1.1E-08	3.0E-06
vesicle-mediated transport	141	2.1E-08	3.8E-06
synaptic vesicle transport	28	2.8E-08	4.8E-06
regulation of synaptic plasticity	38	3.4E-08	5.7E-06
regulation of neurotransmitter levels	42	4.1E-08	6.5E-06
metal ion transport	102	4.3E-08	6.6E-06
postsynaptic membrane	52	3.4E-07	7.0E-06
cell fraction	237	3.5E-07	7.0E-06
cytoplasmic vesicle membrane	47	3.9E-07	7.6E-06
postsynaptic density	40	4.3E-07	8.1E-06
cognition	91	8.2E-08	1.2E-05
neuron projection morphogenesis	70	8.6E-08	1.2E-05
regulation of system process	88	1.0E-07	1.4E-05

**Table 2:** Top GO terms enriched in 1941 *Spalax* genes significantly (*P* value<0.05) up-regulated in muscle/normoxia vs. brain/normoxia.

Term	Count	*P* value	FDR
mitochondrion	364	4.5E-41	2.5E-38
mitochondrial part	175	1.1E-26	3.1E-24
generation of precursor metabolites and energy	92	4.9E-23	1.7E-19
mitochondrial lumen	82	1.9E-20	3.7E-18
mitochondrial matrix	82	1.9E-20	3.7E-18
myofibril	53	8.2E-19	9.4E-17
contractile fiber part	50	1.1E-18	1.1E-16
contractile fiber	55	8.1E-19	1.2E-16
energy derivation by oxidation of organic compounds	54	2.3E-18	3.9E-15
sarcomere	45	9.9E-17	7.9E-15
mitochondrial membrane	117	6.8E-17	9.0E-15
mitochondrial envelope	123	2.3E-16	1.4E-14
mitochondrial inner membrane	100	3.1E-16	1.9E-14
organelle inner membrane	102	3.2E-15	1.7E-13
envelope	150	8.1E-14	3.9E-12
organelle envelope	150	8.1E-14	3.9E-12
acetyl-CoA metabolic process	28	9.9E-15	1.1E-11
mitochondrial membrane part	41	3.2E-13	1.4E-11
coenzyme metabolic process	59	3.6E-14	3.1E-11
ribosome	56	1.3E-12	5.3E-11
coenzyme catabolic process	22	1.2E-13	6.6E-11
cofactor metabolic process	68	1.2E-13	8.0E-11
translation	79	3.7E-13	1.8E-10
acetyl-CoA catabolic process	21	5.6E-13	2.4E-10
cellular respiration	36	9.0E-13	3.1E-10
aerobic respiration	23	8.6E-13	3.3E-10
tricarboxylic acid cycle	20	2.7E-12	8.5E-10
cofactor catabolic process	23	3.8E-12	1.1E-09
I band	30	3.1E-11	1.2E-09
structural constituent of ribosome	45	1.3E-12	1.6E-09
glucose metabolic process	50	3.7E-11	9.8E-09
coenzyme binding	57	5.0E-10	2.9E-07
cofactor binding	70	8.4E-10	3.3E-07
proteasome complex	27	2.0E-08	6.7E-07
Z disc	24	1.9E-08	6.8E-07
structural molecule activity	79	2.5E-09	7.5E-07
translational elongation	29	6.2E-09	1.5E-06
oxidation reduction	107	9.2E-09	2.1E-06
ribosomal subunit	29	8.0E-08	2.5E-06
muscle contraction	33	2.1E-08	4.6E-06
muscle system process	36	2.9E-08	5.9E-06
hexose metabolic process	52	3.1E-08	5.9E-06
sarcolemma	29	9.2E-07	2.7E-05
fatty acid metabolic process	50	3.3E-07	6.0E-05
oxidoreduction coenzyme metabolic process	22	3.7E-07	6.4E-05
mitochondrial respiratory chain	18	2.3E-06	6.6E-05
cytosolic ribosome	19	2.9E-06	7.8E-05
cytosolic part	34	4.1E-06	1.1E-04
dicarboxylic acid metabolic process	20	1.0E-06	1.6E-04
anaphase-promoting complex-dependent proteasomal ubiquitin-dependent protein catabolic process	24	1.3E-06	2.0E-04
ubiquitin-dependent protein catabolic process	50	1.6E-06	2.3E-04
monosaccharide metabolic process	54	1.6E-06	2.3E-04
glycolysis	18	2.7E-06	3.7E-04
ribonucleoprotein complex	79	1.6E-05	3.9E-04
pyruvate metabolic process	19	3.1E-06	4.1E-04
structural constituent of muscle	11	2.5E-06	5.9E-04

**Table 3:** Top GO terms enriched for 314 *Spalax* genes significantly (*P* value<0.05) up-regulated in muscle/hypoxia vs. muscle/normoxia.

Term	Count	P value	FDR
**signal**	37	**2.0E-06**	**2.8E-04**
**glycoprotein**	44	**1.2E-06**	**3.5E-04**
**disulfide bond**	31	**6.2E-06**	**6.0E-04**
**extracellular region**	29	**1.2E-05**	**1.8E-03**
**signal peptide**	37	**4.1E-06**	**2.7E-03**
**extracellular matrix**	15	**1.0E-05**	**3.2E-03**
**glycosylation site:N-linked (GlcNAc…)**	40	**1.1E-05**	**3.4E-03**
**proteinaceous extracellular matrix**	13	**5.1E-05**	**4.0E-03**
**amino acid transmembrane transporter activity**	7	**1.9E-05**	**9.6E-03**
**plasma membrane**	58	**1.6E-04**	**9.9E-03**
**disulfide bond**	28	**6.3E-05**	**1.4E-02**
**vasculature development**	17	**2.8E-05**	**2.7E-02**
**amine transmembrane transporter activity**	7	**1.3E-04**	**3.2E-02**
**transmembrane**	52	**4.8E-04**	**3.4E-02**
**amino-acid transport**	5	**7.2E-04**	**4.1E-02**
**face development**	6	**1.0E-04**	**4.8E-02**
**regulation of cell proliferation**	29	**2.8E-05**	**5.2E-02**
**blood vessel development**	16	**9.5E-05**	**5.9E-02**
**blood vessel morphogenesis**	13	**2.5E-04**	**7.8E-02**
**head development**	6	**2.2E-04**	**8.0E-02**
**regulation of catabolic process**	11	**3.2E-04**	**8.4E-02**
Secreted	14	**2.7E-03**	1.2E-01
extracellular matrix	7	**3.3E-03**	1.3E-01
cell membrane	22	**4.5E-03**	1.5E-01
regulation of cellular catabolic process	9	**7.0E-04**	1.6E-01
intrinsic to membrane	62	**3.3E-03**	1.6E-01
integral to membrane	59	**4.0E-03**	1.6E-01
transcription regulation	24	**5.7E-03**	1.7E-01
growth factor binding	8	**1.3E-03**	1.9E-01
**response to oxygen levels**	15	**1.1E-03**	2.0E-01
response to hormone stimulus	24	**1.1E-03**	2.2E-01
membrane raft	10	**6.4E-03**	2.2E-01
amine transport	8	**1.7E-03**	2.4E-01
**response to hypoxia**	14	**2.1E-03**	2.5E-01
transmembrane receptor protein tyrosine kinase signaling pathway	12	**2.0E-03**	2.5E-01
intrinsic to plasma membrane	12	**8.5E-03**	2.6E-01
enzyme linked receptor protein signaling pathway	15	**1.7E-03**	2.6E-01
platelet-derived growth factor receptor signaling pathway	5	**2.8E-03**	2.6E-01
response to peptide hormone stimulus	14	**2.7E-03**	2.6E-01
regulation of glucose metabolic process	7	**3.4E-03**	2.7E-01
regulation of cell migration	12	**3.0E-03**	2.7E-01
palate development	6	**3.5E-03**	2.7E-01
response to endogenous stimulus	25	**3.3E-03**	2.7E-01
skeletal system development	12	**2.6E-03**	2.7E-01
amino acid transport	7	**2.5E-03**	2.8E-01
response to organic substance	34	**4.0E-03**	2.8E-01
regulation of carbohydrate metabolic process	7	**4.3E-03**	2.8E-01
regulation of cellular carbohydrate metabolic process	7	**4.3E-03**	2.8E-01
regulation of locomotion	12	**4.6E-03**	2.9E-01
negative regulation of cell proliferation	13	**4.2E-03**	2.9E-01
response to steroid hormone stimulus	14	**5.4E-03**	3.2E-01
response to corticosteroid stimulus	9	**5.8E-03**	3.3E-01
response to glucocorticoid stimulus	9	**5.8E-03**	3.3E-01
extracellular space	12	**1.3E-02**	3.4E-01
regulation of epithelial cell differentiation	5	**6.5E-03**	3.4E-01
cell surface receptor linked signal transduction	24	**6.4E-03**	3.5E-01
face morphogenesis	4	**6.9E-03**	3.5E-01
extracellular matrix part	7	**1.6E-02**	3.6E-01
hexose metabolic process	13	**7.6E-03**	3.7E-01

### Predicted proteins

Local multiple alignments of 10,141 non-redundant isotigs (i.e., unique isotigs mapped to non-overlapping genomic target regions) and their mouse/rat/human homologous transcripts were built using the Mafft and Muscle programs. For 9,151 alignments, the coding regions of mouse, rat, and human, overlapped with uninterrupted *S. galili* ORFs. In 4,750 alignments, more than 75% of the coding region of the mouse transcript was aligned to the *S. galili* ORF, indicating that these isotigs may represent the full, or nearly full length *Spalax* coding region (Figure S3a). Note that in many cases *S. galili* predicted ORF segments were significantly larger than the actual alignment size. Although in most cases the entire *S. galili* ORF region was aligned to the target coding region, in some cases large ORF regions were found to have no homology to mouse/rat/human genomes and transcripts. Such cases may potentially represent novel transcript regions or possibly novel genes. In addition different types of insertions and deletions of smaller sizes were found to be specific to *S. galili*.

In order to check *S. galili* predicted ORF phylogeny, a consensus tree was built using 2958 phylogenetic trees based on *S. galili* non-overlapping isotigs with the largest alignments to ORF regions of mouse, rat, cavia, human, marmoset, rhesus , cow, dog, and horse (Figure S3b). Alignments included in this analysis were built only for isotigs mapped uniquely to all tested targets (e.g., Figure 1 for mouse), and only if the mapped genes share the same orthologous group in all tested species (see Figure 2A, however for 5 target species). As expected, *Spalax* is found to be located in the *Muroide*a superfamiliy within the *Rodentia* clade (Figure S3b) indicating the isotigs are phylogenetically informative. The same tree structure was obtained for larger sets of alignments to ORFs (∼5000, ∼7000 alignments), however the number of trees with consensus partitions was approximately 20% lower in the cavia/Muroidea and Laurasiatheria/ Euarchontoglires nodes, most likely because alignments of small sizes were less informative.

### Single nucleotide polymorphism

Single nucleotide polymorphism (SNPs) alleles were identified as single nucleotide replacements in overlapping reads, within an isotig. SNP containing regions with read-depth <6 were filtered out and alleles occurring in <30% of the overlapping reads were excluded. In order to reduce false-positive results that reflect low sequencing quality candidate SNPs flanked by nucleotides with ambiguous identity were filtered out. For all isotigs mapped to a single gene (e.g. predicted alternatively spliced transcripts) the one with the largest number of detected SNPs was selected. Based on these criteria a total of 5,746 non-redundant SNPs were found in 3,340 predicted genes. Out of these, 4,215, 867, 295, and 369 SNPs, represented A↔G/C↔T, A↔C/G↔T, A↔T, and G↔C nucleotide replacements, respectively (73% transition events). After excluding SNP containing regions with read depths <31, a total of 1000 SNPs remained (with an increase in the percentage of transitions to 79%).

### Identification of *S. galili* non-conserved transcript regions

**Figure 3:**
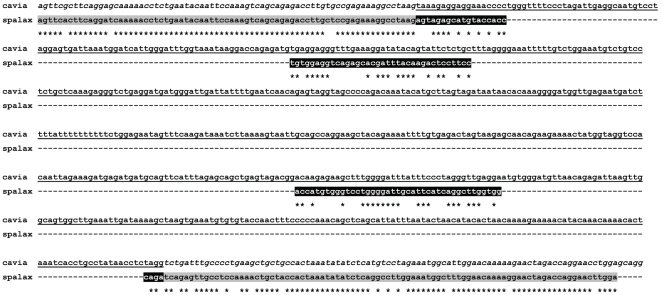
Pairwise alignment of *S. galili* transcribed insert against the homologous *Cavia porcellus* melusin genomic region. In order to validate Blast and Lastz indications that the *S. galili* novel transcribed regions are non-conserved in target genomes we locally aligned the *Spalax* insert as well as the conserved flanking regions to the target genome. (see Methods). An example is shown above for the putative novel transcribed region in the *Spalax melusin* transcript (isotig19920). The highly conserved flanking regions (gray blocks) mapped to target regions harboring *Cavia* exons 4 and 5. The novel *Spalax* transcribed region (black blocks) is only weakly aligned to the *Cavia* intronic region, indicating that a homologous exon is probably missing in the target genomic region. Similar alignments were constructed for different target mammals in order to decrease the probability of false positive detection of novel exons. The Cavia exons are labeled in italics. The Cavia intron is underlined.

We identified novel *S. galili* exons with no evidence for conservation beyond S. *ehrenbergi* based on similarity searches against mammalian genomes and transcriptomes. Two types of novel regions were identified: (1) *S. galili* non-conserved exons (Figure 3); (2) *S. galili* insertions located inside predicted conserved exons far from predicted exon boundaries. Details and properties of these regions are shown in Table S4. Multiple sequence alignments of these regions against target transcripts are shown in Figure S4. Several interesting examples of putative novel ORF regions were experimentally verified in *S. galili*, and found to be missing in rat, based on RT-PCR with cDNA templates from brain and muscle tissues of rat and *S. galili* (Table S4). In addition, different putatively non-conserved transcribed regions were found to include premature termination codons (PTC). The 5′/3′ ends of these regions were frequently found proximal to splice-junctions. Though we cannot exclude the possibility that most of these PTC+ regions result from intron contamination, several of these regions were tested using RT-PCR on *S. galili* cDNAs and exhibited high transcription levels with no amplification in rat cDNAs. Numerous nonsense-mediated mRNA decay degraded PTC+ variants were previously found in mouse and most were non-conserved in mouse vs. human [48].

**Figure 4:**
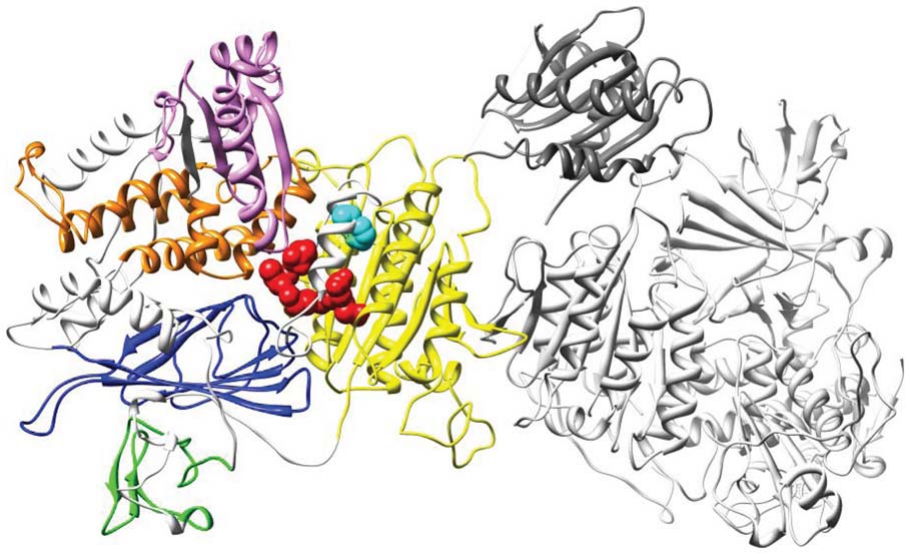
A model of the *S. galil sec23a/sec24* hetero-dimer, color-coded by functional domains. The protein *sec23a* appears on the left in bright colors in its putative docked conformation with its *sec24* partner (white and grey chains on the right). The 5 known domains are colored in yellow (trunk domain), green (zinc finger domain), violet (Gelsolin), orange (helical domain), and blue (beta-sandwich domain). Putative novel *S. galili* splice variant flanking amino acids (the start and end point of the new insertion) are presented in red spheres (the novel region is not modeled), and the F382L mutation in cyan spheres.

In order to estimate potential structural/functional significance of these novel regions within proteins, we attempted to predict protein structures of *S. galili* genes with putative novel insertions. Predicted protein sequences of two *S. galili* non-conserved transcript variants, *sec23a*, and *TPPII* (Table S4), were structurally aligned to known homologous proteins (see Methods). In all other examples (Table S4), no available templates had enough similarity to build structures including the novel sequences. In the case of *sec23a*, *S. galili* and human (PDB code 2nut, chain A) predicted proteins have 99% identity, and therefore an accurate model was built. Interestingly, the *S. galili* putative novel region of this transcript was found to be spatially very close to a known deleterious F382L mutation in human, Cranio-lenticulo-sutural dysplasia, characterized by changes of skull and face morphology. This novel *S. galili* exon of *sec23a* is located between the trunk domain, and the beta-sandwich domain [49] which also includes the critical F382L mutation mentioned above. The 3D model shows that the variant region is located within a central position on the structure with probable important consequences to the protein function and structure (Figure 4). In the case of *TPPII S. galili* and drosophila (PDB code 3lxu), the template protein has 38% identity and the novel *S. galili* region was found to be located in the hinge region of the protein far from both the dimer interface or the enzymatic reaction center which suggests a primary structural impact of this insertion [50].

## Discussion

The evolutionary emergence of subterranean species, and among them *S. ehrenbergi*, is associated with multiple changes in hypoxia-induced pathways, visual/somatosensory/motor/auditory centers in the brain, cardiovascular systems, skeletal morphology, and other pathways and systems. Different molecular studies have begun to unravel the complex connection between physiological and molecular mechanisms related to S. *ehrenbergi* evolution. Yet, what we know about *S. ehrenbergi* in terms of molecular evolution is still based on a very limited number of genes. Here we report *S. galili* transcriptome sequencing representing more than 11,000 predicted genes as a first step in a genome-wide search for *S. ehrenbergi* adaptive genes. These transcript regions were expressed in muscle and brain tissues under hypoxia and normoxia and sequenced using Sanger, GS FLX, and FLX Titanium technologies.

### *Spalax* novel transcribed regions

Different studies have compared the properties of *S. ehrenbergi* genes to those of related rodents (e.g., rat) in an attempt to identify specific selective changes that emerged during *S. ehrenbergi* or *Spalacidae* evolution. For example, recent studies suggested that non-conserved splice-variants of the gene *heparanase* in *S. ehrenbergi* may contribute to the adaptation of this species to hypoxic environments [51], [52]. Similarly, we have detected *S. galili* non-conserved transcribed regions with possible evolutionary importance. Our working hypothesis was that the emergence of novel transcribed regions in *S. ehrenbergi*, especially within conserved coding regions, may reflect positive selection of novel functional elements. Several hundreds of such regions with more than 50 bp length and with no apparent orthology to known transcripts were detected. Among these regions we discovered ∼30 segments of ∼50–400 bp residing in putative coding regions as part of ORFs with no termination codons (for examples, see Table S4, Figure S4). Though each of these putative non-conserved regions only negligibly contributes to the overall complexity of its gene, the emergence of these regions within S. *ehrenbergi* relatively recent evolution is intriguing. Several of these putatively novel ORF regions reside in genes that may be important for protection against hypoxia stress, for example genes involved in heart hypertrophy, or DNA repair [53]. The presence/absence of several of these non-conserved transcribed regions in *S. galili* as compared to rat cDNAs was also verified using RT-PCR (see Table S4).

We detected a highly polymorphic, large non-conserved coding tri-nucleotide microsatellite insertion in the *S. galili CINAP* gene, which is known to be involved in the regulation of the *NR2b* subunit of the *NMDA* receptor, a critical subunit in the protection against hypoxia damage in the brain [54], [55]. This insertion is mapped proximal to a PEST domain in mouse *CINAP*, which may be involved in the regulation of *NR2b*. An additional example is the *S. galili SEC23a* gene which includes an alternatively spliced region mapped to a human intron, and at the protein 3D level, proximal to the active center (see Figure 4). In human, this gene is thought to be crucial for the morphogenesis of the skull and face [56]. As previous studies suggested the evolutionary emergence of novel transcribed regions may potentially underlie the phenotypic divergence between species [57]–[59]. Accordingly, it seems that further investigation of such novel transcript regions, especially unique substitutions and indels in positions mapped to functional domains in proteins, may contribute to the understanding of *S. ehrenbergi* molecular evolution.

Extensive transcriptome plasticity identified from the isotigs is reflected by the presence of multiple combinations of exons in splice variants. The tested isotigs are an integral part of the Newbler contig/isotig/isogroup hierarchy, which theoretically reflects the relationships between transcript regions, transcript variants and genes, respectively. Multiple isotigs were classified as variants of specific genes employing homology based annotations.

The isotigs identified in this work may be used to search for genes which have lost or modified their original functions during *S. ehrenbergi* evolution, such as genes involved in visual perception, repair of UV induced DNA damage, and light sensitive molecules. An intriguing question is whether such molecules and pathways are degenerate, or alternatively, acquired novel functions as a result of relaxation of selective pressure. As isotig ORFs are phylogenetically informative (see Figure S3) it is now possible to test these hypotheses. In addition, as it was suggested that the speciation of subterranean mammals was a rapid occurrence from an evolutionary perspective [60], it is intriguing whether this hypothesis could be validated from the comparative data obtained in this study.

### Differential gene expression inferred from the *Spalax* transcriptome

As GS FLX Titanium isotigs were assembled from four different sources (brain/normoxia, brain/hypoxia, muscle/normoxia, and muscle/hypoxia), we tested whether the proportions of read counts originating from different sources may reflect differential expression. The results indicate a highly significant over-representation of brain and muscle related GO terms, from the isotigs with the largest proportions of reads in brain and muscle tissues, respectively. Nevertheless, as expected, we observed fewer differences in read counts between muscle-hypoxia vs. muscle-normoxia, and brain-hypoxia vs. brain-normoxia. These limited differences may reflect the efficiency of the cDNA normalization procedure for most genes expressed within the same tissues, even under different treatments.

Among the terms enriched in genes up-regulated in muscle-hypoxia vs. muscle-normoxia (Table 3), “vasculature development”, “blood vessel development and/or morphogenesis”, “proteinaceous extracellular matrix”, and “extracellular matrix”, may reflect activation of processes related to the sprouting of endothelial cells, and cell migration/invasion, in hypoxia. The enrichment of proteins capable of disulfide bond formation is interesting. These bonds are formed by the oxidation of the thiol groups in cysteins, and are disassociated when the cellular oxygen pressure is diminished, or in the presence of reducing agents, thereby leading to changes in protein conformation and activity. Moreover, the sensitivity of disulfide bonds to oxygen pressure can be utilized for oxidative-stress sensing, in the presence of NO, as demonstrated in *NMDAR* in the brain and in *RyR1* channels in muscles [61], [62]. Though not significant after FDR correction, the appearance of the expected terms “response to oxygen levels” and “response to hypoxia” among the terms with the highest enrichment should also be noted. Interestingly, the *Spalax ankyrin repeat domain 1 (Ankrd1)* gene which was previously identified with an unusual 40–50 fold increase in expression during hypoxia in *Spalax* [63] is found in the current study among the 10 genes with the largest estimated differences in read counts for muscle/hypoxia vs. muscle/normoxia (Table S2). Similarly, additional genes previously found to be over-expressed in hypoxia using microarray data [47] have also exhibited the same trend here (Table S2). Furthermore, among the group of genes with the most significant up-regulation in hypoxia many are known to be involved in processes related to hypoxia and oxidative stress response in other mammals, as elaborated in Table S5. Therefore, despite the limitations, the current read count data may be further used for identification of alternatively expressed gene candidates in S. *ehrenbergi*, especially for genes exhibiting strong differences between tissues/treatment. One of the main advantages of the data presented here, for this purpose, is that it allows estimating differences in the abundance of transcript variants in different tissues/treatments, based on read-depth proportions in different positions along isotigs and not only information about estimated transcription levels per gene.

### Achievements and utility of *Spalax* transcriptome sequencing

A total of 51,885 isotigs with an average length of 1,588 bp were assembled based on reads produced in all runs. Isotigs uniquely mapped to the same reference genes were clustered in 12,107, 11,406, and 12,074 clusters, containing 18,022, 15,317, and 18,518 non-overlapping isotigs for mouse, rat, and human references, respectively (see Figure S1). In 8,774 of 12,107 mouse-based clusters, the isotigs with the largest HSPs portion were mapped to mouse/rat/human genes known to be part of the same orthologous group (Figure 2). For the remaining clusters, the isotigs with the largest HSPs were mapped to mouse/human/rat genes that were not part of the same orthologous group. It was possible to show that this inconsistency is partly attributed to very short isotigs, and/or isotigs representing weakly conserved regions such as 3′ UTRs (see Figure 2). In addition, the ambiguity of isotig annotations may reflect assembly errors, similarity to paralogous genes, and cases of genes with unknown orthologous counterparts in at least one of the target species.

In order to investigate the coding portion of the sequences, 9,151 non-redundant isotigs that harbor predicted ORFs were locally aligned to homologous mouse/rat/human transcripts, and 4,750 of them were found to represent a full or close to full coding portion of mRNA (see Figure S3). The forgoing ORFs could be highly valuable for the detection of *S. ehrenbergi* positively selected coding regions. In addition, ∼5,700 non-redundant novel SNPs were detected creating a valuable resource for future genetic studies in *S. ehrenbergi* and for analyses of transcriptome divergence within S. *ehrenbergi* populations and families. Such analyses may be very important for species-conservation and population-genetics studies in *S. ehrenbergi* since population survival of this species complex may depend on its genetic diversity and inbreeding avoidance, specifically for isolated/sub-structured populations. In addition, *S. ehrenbergi* genetic diversity is reflected by large changes in genetic variability and selection regimes between different populations, chromosomal types, and ecologies [64].

## Supporting Information

Figure S1 **Homology-based annotation pipeline.**
(DOC)

Figure S2 **Differential expression in brain vs. muscle under normoxia (left), and muscle under hypoxia vs. normoxia (right).** *M* (vertical axis) represents reads count differences, and is equal to log_2_ *^R1^*−log_2_ *^R2^*, where *R1* and *R2* are the read counts for two groups of reads mapped to the same gene. *A* (horizontal axis) represents average intensity of expression, and is equal to (log_2_ *^R1^*+log_2_ *^R2^*)/2. Dots around the median distribution of *M* values (i.e., *M* = −1.5 in the plots), represent genes expressed at similar proportions between libraries, such as housekeeping genes. The median distribution of *M* values≠0, because of large differences in the total reads between libraries. Red dots represent genes identified as differentially expressed with random sampling model (see Methods).
(DOC)

Figure S3 **Analysis of ORFs.** (**A**) The number of non-redundant *S. galili* predicted ORFs as a function of aligned ORF size (#codons) based on local multiple alignments of isotigs to mouse/rat/human full coding regions. The bars represent the number of non-redundant isotigs harboring uninterrupted ORFs<75% (blue bars) or ≥75% (red bars) of the full coding region of the homologous mouse transcript. In 4750 out of 9151 ORFs>75% of the full coding region of the reference transcript was aligned to the *S. galili* ORF. (**B**) Consensus tree was built using phylogenetic trees based on multiple nucleotide sequence alignments of 2985 *S. galili* predicted genes with the largest alignments to orthologous ORFs of mouse, rat, cavia (rodentia clade, Euarchontoglires superorder, in blue), human, marmoset, rhesus (primates clade, Euarchontoglires superorder, in red), cow, dog, and horse (Laurasiatheria superorder, green). The labels near the branches show the number of cases where a partition occurred out of 2985 cases. The same tree structure was found based on alignments of ∼7000 ORFs>50 codons but with lower partition stability.
(DOC)

Figure S4 **Alignments of** ***S. galili*** **novel transcribed regions to their putative orthologous transcripts of mouse, rat, and human: (S)** ***S. galili*** **, (M) mouse, (R) rat, (H) human.** The novel transcribed regions were translated to predicted proteins based on the protein sequences derived from their orthologous transcripts (black blocks). In the genes *Rpl4* and *Rtn3*, repeats within the novel region are shown as tandemly repeated black and gray blocks.
(DOC)

Table S1 **BLASTX and BLASTN best hits for Spalax contigs and isotigs.**
(XLSX)

Table S2 **Top 50 genes with the most significant differences in read counts for** ***Spalax*** **muscle/hypoxia vs. muscle/normoxia.** The titles ‘#n’, and ‘#h’, denote: number of reads in muscle/normoxia vs. muscle/hypoxia libraries. Note that the total number of reads assembled from muscle/hypoxia (C.4) is about 2.4 times larger than those from muscle/normoxia (C.3). The conducted differential expression test (see Methods) takes into account the **global** difference in read counts between different tissues/treatments. Gene names in black bold fonts designate genes that were previously identified as differentially expressed by microarray data. Gene names in gray bold fonts indicate that differentially expressed isozymes or paralogs were found previously. FDR column denotes adjusted *P* values.
(DOC)

Table S3 **The titles ‘#n’, and ‘#h’, denote: number of reads in brain/normoxia vs. brain/hypoxia libraries.** Note that the total number of reads assembled from brain/hypoxia (C.2) is about 1.8 times larger than those from brain/normoxia (C.1). The conducted differential expression test (see Methods) takes into account the global difference in read counts between different tissues/treatments.
(DOC)

Table S4 **Examples of non-conserved/weakly-conserved** ***S. galili*** **transcribed regions.** Column ‘Repeat’: Repeat Masker output for the non-conserved *Spalax* region; Columns ‘*Spalax*’/’rat’: electrophoresis and sequencing results of RT-PTR products (where ‘√’ and ‘×’ indicate that the expected band was detected/not detected in cDNAs, blank cells denote untested cases; ORF/PTC+ indicate whether the tested novel transcribed regions are part of an ORF or contain a termination codon). ‘*’ indicates that though the tested transcribed region was not found experimentally in rat it shows weak similarity to introns of some target species, but not to known exons. Size: denotes novel region size in bp.
(DOC)

Table S5 **Known functional roles of genes most significantly up-regulated in muscle/hypoxia vs. muscle/normoxia (C.4 vs. C.3) and brain/hypoxia vs. brain/normoxia (C.2 vs. C.1).** The genes shown in this table are taken from the entire list of genes up-regulated in muscle/hypoxia vs. muscle/normoxia (table S1) and brain/hypoxia vs. brain/normoxia (table S2).
(DOC)

## References

[1] Shams I, Avivi A, Nevo E (2005). Oxygen and carbon dioxide fluctuations in burrows of subterranean blind mole rats indicate tolerance to hypoxic-hypercapnic stresses.. Comp Biochem Physiol A Mol Integr Physiol.

[2] Arieli R, Nevo E (1991). Hypoxic survival differs between two mole rat species (*Spalax ehrenbergi*) of humid and arid habitats.. Comp Biochem Physiol A Comp Physiol.

[3] Widmer HR, Hoppeler H, Nevo E, Taylor CR, Weibel ER (1997). Working underground: Respiratory adaptations in the blind mole rat.. Proc Natl Acad Sci U S A.

[4] Avivi A, Resnick MB, Nevo E, Joel A, Levy AP (1999). Adaptive hypoxic tolerance in the subterranean mole rat *Spalax ehrenbergi*: The role of vascular endothelial growth factor.. FEBS Lett.

[5] Arieli R, Ar A (1981). Heart rate responses of the mole rat (*Spalax ehrenbergi*) in hypercapnic, hypoxic, and cold conditions.. Physiol Zool.

[6] Storier D, Wollberg Z, Ar A (1981). Low and nonrhythmic heart rate of the mole rat (*Spalax ehrenbergi*): Control by the autonomic nervous system.. Journal of Comparative Physiology B: Biochemical, Systemic, and Environmental Physiology.

[7] Nevo E, Ben-Shlomo R, Maeda N (1989). Haptoglobin DNA polymorphism in subterranean mole rats of the *Spalax ehrenbergi* superspecies in israel.. Heredity.

[8] Gurnett AM, O'Connell JP, Harris DE, Lehmann H, Joysey KA (1984). The myoglobin of rodents: Lagostomus maximus (viscacha) and *Spalax ehrenbergi* (mole rat).. J Protein Chem.

[9] Kleinschmidt T, Nevo E, Braunitzer G (1984). The primary structure of the hemoglobin of the mole rat (*Spalax ehrenbergi*, rodentia, chromosome species 60).. Hoppe-Seyler' s Zeitschrift Für Physiologische Chemie.

[10] Hankeln T, Ebner B, Fuchs C, Gerlach F, Haberkamp M (2005). Neuroglobin and cytoglobin in search of their role in the vertebrate globin family.. J Inorg Biochem.

[11] Avivi A, Gerlach F, Joel A, Reuss S, Burmester T (2010). Neuroglobin, cytoglobin, and myoglobin contribute to hypoxia adaptation of the subterranean mole rat *Spalax*.. Proc Natl Acad Sci U S A.

[12] Gerlach F, Avivi A, Joel A, Burmester T, Nevo E (2006). Genomic organization and molecular evolution of the genes for neuroglobin and cytoglobin in the hypoxiatolerant israeli mole rat, *Spalax* carmeli.. Israel Journal of Ecology and Evolution.

[13] Shams I, Avivi A, Nevo E (2004). Hypoxic stress tolerance of the blind subterranean mole rat: Expression of erythropoietin and hypoxia-inducible factor 1 alpha.. Proc Natl Acad Sci U S A.

[14] Shams I, Nevo E, Avivi A (2005). Ontogenetic expression of erythropoietin and hypoxia-inducible factor-1 alpha genes in subterranean blind mole rats.. FASEB J.

[15] Ashur-Fabian O, Avivi A, Trakhtenbrot L, Adamsky K, Cohen M (2004). Evolution of p53 in hypoxia-stressed *Spalax* mimics human tumor mutation.. Proc Natl Acad Sci U S A.

[16] Avivi A, Ashur-Fabian O, Joel A, Trakhtenbrot L, Adamsky K (2007). P53 in blind subterranean mole rats–loss-of-function versus gain-of-function activities on newly cloned *Spalax* target genes.. Oncogene.

[17] Band M, Ashur-Fabian O, Avivi A (2010). The expression of p53-target genes in the hypoxia-tolerant subterranean mole-rat is hypoxia-dependent and similar to expression patterns in solid tumors.. Cell Cycle.

[18] Avivi A, Resnick MB, Nevo E, Joel A, Levy AP (1999). Adaptive hypoxic tolerance in the subterranean mole rat *Spalax ehrenbergi*: The role of vascular endothelial growth factor.. FEBS Lett.

[19] Avivi A, Shams I, Joel A, Lache O, Levy AP (2005). Increased blood vessel density provides the mole rat physiological tolerance to its hypoxic subterranean habitat.. FASEB J.

[20] Jubb AM, Landon TH, Burwick J, Pham TQ, Frantz GD (2003). Quantitative analysis of colorectal tissue microarrays by immunofluorescence and in situ hybridization.. J Pathol.

[21] Jubb AM, Pham TQ, Hanby AM, Frantz GD, Peale FV (2004). Expression of vascular endothelial growth factor, hypoxia inducible factor 1alpha, and carbonic anhydrase IX in human tumours.. J Clin Pathol.

[22] Frahm HD, Rehkamper G, Nevo E (1997). Brain structure volumes in the mole rat, *Spalax ehrenbergi* (spalacidae, rodentia) in comparison to the rat and subterrestrial insectivores.. J Hirnforsch.

[23] Catania KC, Remple MS (2002). Somatosensory cortex dominated by the representation of teeth in the naked mole-rat brain.. Proc Natl Acad Sci U S A.

[24] Necker R, Rehkamper G, Nevo E (1992). Electrophysiological mapping of body representation in the cortex of the blind mole rat.. Neuroreport.

[25] Cooper HM, Herbin M, Nevo E (1993). Ocular regression conceals adaptive progression of the visual system in a blind subterranean mammal.. Nature.

[26] Nevo E (1999). Mosaic evolution of subterranean mammals: Regression, progression, and global convergence.

[27] Ben-Shlomo R, Ritte U, Nevo E (1995). Activity pattern and rhythm in the subterranean mole rat superspecies *Spalax ehrenbergi*.. Behav Genet.

[28] Avivi A, Albrecht U, Oster H, Joel A, Beiles A (2001). Biological clock in total darkness: The Clock/MOP3 circadian system of the blind subterranean mole rat.. Proc Natl Acad Sci U S A.

[29] Avivi A, Oster H, Joel A, Beiles A, Albrecht U (2002). Circadian genes in a blind subterranean mammal II: Conservation and uniqueness of the three period homologs in the blind subterranean mole rat, *Spalax ehrenbergi* superspecies.. Proc Natl Acad Sci U S A.

[30] Hough RB, Avivi A, Davis J, Joel A, Nevo E (2002). Adaptive evolution of small heat shock protein/alpha B-crystallin promoter activity of the blind subterranean mole rat, *Spalax ehrenbergi*.. Proc Natl Acad Sci U S A.

[31] Dassanayake M, Haas JS, Bohnert HJ, Cheeseman JM (2009). Shedding light on an extremophile lifestyle through transcriptomics.. New Phytol.

[32] Milne I, Bayer M, Cardle L, Shaw P, Stephen G (2010). Tablet–next generation sequence assembly visualization.. Bioinformatics.

[33] Gordon D (2003). Viewing and editing assembled sequences using consed.. Curr Protoc Bioinformatics Chapter.

[34] Altschul SF, Gish W, Miller W, Myers EW, Lipman DJ (1990). Basic local alignment search tool.. J Mol Biol.

[35] Fujita PA, Rhead B, Zweig AS, Hinrichs AS, Karolchik D (2011). The UCSC genome browser database: Update 2011.. Nucleic Acids Res.

[36] Schwartz S, Kent WJ, Smit A, Zhang Z, Baertsch R (2003). Human-mouse alignments with BLASTZ.. Genome Res.

[37] Katoh K, Asimenos G, Toh H (2009). Multiple alignment of DNA sequences with MAFFT.. Methods Mol Biol.

[38] Guindon S, Gascuel O (2003). A simple, fast, and accurate algorithm to estimate large phylogenies by maximum likelihood.. Syst Biol.

[39] Felsenstein J (1989). PHYLIP-phylogeny inference package (version 3.2).. Cladistics.

[40] Soding J, Biegert A, Lupas AN (2005). The HHpred interactive server for protein homology detection and structure prediction.. Nucleic Acids Res.

[41] Eswar N, Webb B, Marti-Renom MA, Madhusudhan MS, Eramian D (2007). Comparative protein structure modeling using MODELLER.. Curr Protoc Protein Sci Chapter.

[42] Pettersen EF, Goddard TD, Huang CC, Couch GS, Greenblatt DM (2004). UCSF chimera–a visualization system for exploratory research and analysis.. J Comput Chem.

[43] Finn RD, Mistry J, Tate J, Coggill P, Heger A (2010). The pfam protein families database.. Nucleic Acids Res.

[44] Wang L, Feng Z, Wang X, Wang X, Zhang X (2010). DEGseq: An R package for identifying differentially expressed genes from RNA-seq data.. Bioinformatics.

[45] Benjamini Y, Hochberg Y (1995). Controlling the false discovery rate: A practical and powerful approach to multiple testing.. Journal of the Royal Statistical Society.Series B (Methodological).

[46] Storey JD, Tibshirani R (2003). Statistical significance for genomewide studies.. Proc Natl Acad Sci U S A.

[47] Avivi A, Brodsky L, Nevo E, Band MR (2006). Differential expression profiling of the blind subterranean mole rat *Spalax ehrenbergi* superspecies: Bioprospecting for hypoxia tolerance.. Physiol Genomics.

[48] Lareau LF, Brooks AN, Soergel DA, Meng Q, Brenner SE (2007). The coupling of alternative splicing and nonsense-mediated mRNA decay.. Adv Exp Med Biol.

[49] Mancias JD, Goldberg J (2007). The transport signal on Sec22 for packaging into COPII-coated vesicles is a conformational epitope.. Mol Cell.

[50] Chuang CK, Rockel B, Seyit G, Walian PJ, Schonegge AM (2010). Hybrid molecular structure of the giant protease tripeptidyl peptidase II.. Nat Struct Mol Biol.

[51] Nasser NJ, Nevo E, Shafat I, Ilan N, Vlodavsky I (2005). Adaptive evolution of heparanase in hypoxia-tolerant *Spalax*: Gene cloning and identification of a unique splice variant.. Proc Natl Acad Sci U S A.

[52] Nasser NJ, Avivi A, Shafat I, Edovitsky E, Zcharia E (2009). Alternatively spliced *Spalax* heparanase inhibits extracellular matrix degradation, tumor growth, and metastasis.. Proc Natl Acad Sci U S A.

[53] Bristow RG, Hill RP (2008). Hypoxia and metabolism. hypoxia, DNA repair and genetic instability.. Nat Rev Cancer.

[54] Wang GS, Hong CJ, Yen TY, Huang HY, Ou Y (2004). Transcriptional modification by a CASK-interacting nucleosome assembly protein.. Neuron.

[55] Marks JD (2009). Regulation of vulnerability to NMDA excitotoxicity during postnatal maturation.. Brain Hypoxia and Ischemia.

[56] Boyadjiev SA, Fromme JC, Ben J, Chong SS, Nauta C (2006). Cranio-lenticulo-sutural dysplasia is caused by a SEC23A mutation leading to abnormal endoplasmic-reticulum-to-golgi trafficking.. Nat Genet.

[57] Wetterbom A, Ameur A, Feuk L, Gyllensten U, Cavelier L (2010). Identification of novel exons and transcribed regions by chimpanzee transcriptome sequencing.. Genome Biol.

[58] Fondon JW, Garner HR (2004). Molecular origins of rapid and continuous morphological evolution.. Proc Natl Acad Sci U S A.

[59] Fondon JW, Hammock EAD, Hannan AJ, King DG (2008). Simple sequence repeats: Genetic modulators of brain function and behavior.. Trends Neurosci.

[60] Bush GL, Case SM, Wilson AC, Patton JL (1977). Rapid speciation and chromosomal evolution in mammals.. Proc Natl Acad Sci U S A.

[61] Takahashi H, Shin Y, Cho SJ, Zago WM, Nakamura T (2007). A novel thiol oxygen sensor: Hypoxia enhances S-Nitrosylation—Mediated inhibition of NMDA receptor activity.. Neuron.

[62] Eu JP, Sun J, Xu L, Stamler JS, Meissner G (2000). The skeletal muscle calcium release channel: Coupled O2 sensor and NO signaling functions.. Cell.

[63] Band M, Joel A, Avivi A (2010). The muscle ankyrin repeat proteins are hypoxia-sensitive: In vivo mRNA expression in the hypoxia-tolerant blind subterranean mole rat, *Spalax ehrenbergi*.. J Mol Evol.

[64] Nevo E, Ivanitskaya E, Beiles A (2001). Adaptive radiation of blind subterranean mole rats: Naming and revisiting the four sibling species of the *Spalax ehrenbergi* superspecies in Israel: *Spalax galili* (2n = 52), *S. g*olani (2n = 54), S. carmeli (2n = 58), and S. judaei (2n = 60).

