# Supplementary material for: Transcriptome Sequencing of the Blind Subterranean Mole Rat, Spalax galili: Utility and Potential for the Discovery of Novel Evolutionary Patterns
Source: PLoS One. 2011 Aug 12;6(8):e21227. doi: 10.1371/journal.pone.0021227 (PMC3155515; doi:10.1371/journal.pone.0021227)
Supplement: Figure S1 — Homology-based annotation pipeline. (DOC) [file pone.0021227.s001.doc]

| Assembly |  | Blast |  | (fig. 1) |  |  |  | (figs. 2, and 3) |  |  |
| --- | --- | --- | --- | --- | --- | --- | --- | --- | --- | --- |
| 51,855 isotigs  (combined  assembly) |  | 45,700 isotigs were mapped to mouse transcripts |  | 31,715 unique isotigs in 12,107 mouse gene clusters |  | 18,022  non-overlapping isotigs were selected |  | 17,664 non-overlapping isotigs were compared, in mouse, rat, and human based annotations. |  | Category 3 :  11,884 isotigs (8,774 gene clusters) |
|  |  |  |  |  |  |  |  |  |  |  |
|  |  | 43,600 isotigs were mapped to rat  transcripts |  | 29,216 unique  isotigs in 11,406 gene rat clusters |  | 15,317  non-overlapping isotigs were selected |  |  |  | Category 2:  4,061 isotigs (2,106 gene clusters) |
|  |  |  |  |  |  |  |  |  |  |  |
|  |  | 44,500 isotigs were mapped to human transcripts |  | 32,078 unique isotigs in 12,074 human gene clusters |  | 18,518  non-overlapping isotigs were selected |  |  |  | Category 1:  1,719 isotigs (1,016 gene clusters) |
