# Supplementary figures and images for: Transcriptome Sequencing of the Blind Subterranean Mole Rat, Spalax galili: Utility and Potential for the Discovery of Novel Evolutionary Patterns
Source: PLoS One. 2011 Aug 12;6(8):e21227. doi: 10.1371/journal.pone.0021227 (PMC3155515; doi:10.1371/journal.pone.0021227)

**
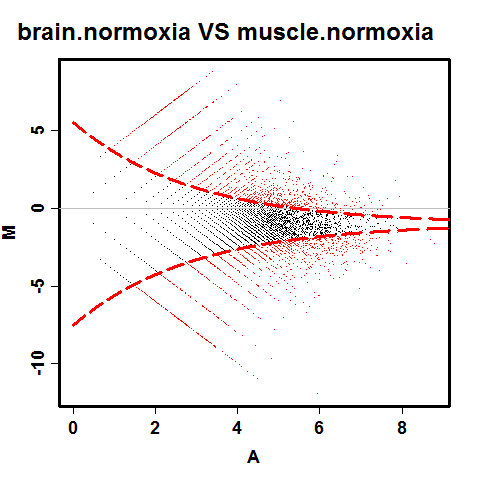
**
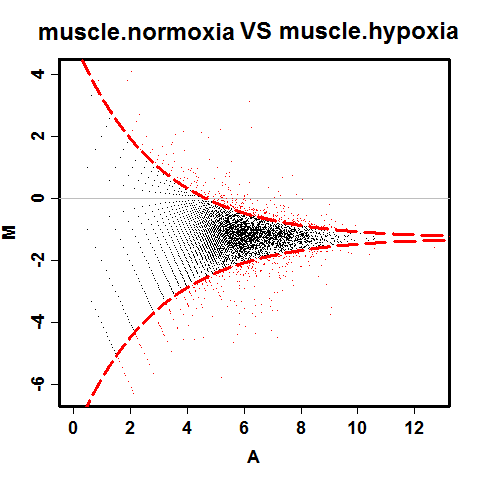

Supplement: Figure S2 — Differential expression in brain vs. muscle under normoxia (left), and muscle under hypoxia vs. normoxia (right). M (vertical axis) represents reads count differences, and is equal to log2 R1−log2 R2, where R1 and R2 are the read counts for two groups of reads mapped to the same gene. A (horizontal axis) represents average intensity of expression, and is equal to (log2 R1+log2 R2)/2. Dots around the median distribution of M values (i.e., M = −1.5 in the plots), represent genes expressed at similar proportions between libraries, such as housekeeping genes. The median distribution of M values≠0, because of large differences in the total reads between libraries. Red dots represent genes identified as differentially expressed with random sampling model (see Methods). (DOC) [file pone.0021227.s002.doc]

**A**


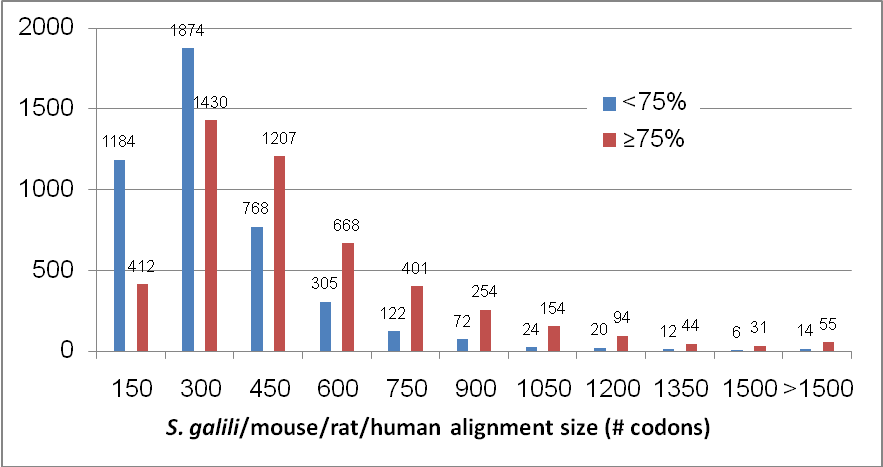


**B**

Supplement: Figure S3 — Analysis of ORFs. (A) The number of non-redundant S. galili predicted ORFs as a function of aligned ORF size (#codons) based on local multiple alignments of isotigs to mouse/rat/human full coding regions. The bars represent the number of non-redundant isotigs harboring uninterrupted ORFs<75% (blue bars) or ≥75% (red bars) of the full coding region of the homologous mouse transcript. In 4750 out of 9151 ORFs>75% of the full coding region of the reference transcript was aligned to the S. galili ORF. (B) Consensus tree was built using phylogenetic trees based on multiple nucleotide sequence alignments of 2985 S. galili predicted genes with the largest alignments to orthologous ORFs of mouse, rat, cavia (rodentia clade, Euarchontoglires superorder, in blue), human, marmoset, rhesus (primates clade, Euarchontoglires superorder, in red), cow, dog, and horse (Laurasiatheria superorder, green). The labels near the branches show the number of cases where a partition occurred out of 2985 cases. The same tree structure was found based on alignments of ∼7000 ORFs>50 codons but with lower partition stability. (DOC) [file pone.0021227.s003.doc]
