# Supplementary material for: Transcriptome Sequencing of the Blind Subterranean Mole Rat, Spalax galili: Utility and Potential for the Discovery of Novel Evolutionary Patterns
Source: PLoS One. 2011 Aug 12;6(8):e21227. doi: 10.1371/journal.pone.0021227 (PMC3155515; doi:10.1371/journal.pone.0021227)
Supplement: Figure S4 — Alignments of S. galili novel transcribed regions to their putative orthologous transcripts of mouse, rat, and human: (S) S. galili , (M) mouse, (R) rat, (H) human. The novel transcribed regions were translated to predicted proteins based on the protein sequences derived from their orthologous transcripts (black blocks). In the genes Rpl4 and Rtn3, repeats within the novel region are shown as tandemly repeated black and gray blocks. (DOC) [file pone.0021227.s004.doc]

*TPPII (isotig20772)*

**S** tgccaacaacggctcttcccagctcctgcctccgggttcctgccttgagttcctgtcctggcttccctcagtgatggattgtgacctgggagttgccaataaactaatcaaggaggagcttcaa

**M** tgccaacaatggctcttcccaa------------------------------------------------------------------------gccaataaactaatcaaggaagagttgcaa

**R** tgccaataatggctcttcccaa------------------------------------------------------------------------gccaataaactaatcaaggaagagttgcag

**H** tgccaacaacggctcttctcaa------------------------------------------------------------------------gcaaataaactaatcaaggaggaacttcaa

****** ** ******** ** ** ***************** ** * **

**S** V..A..N..N..G..S..S..Q..L..L..P..P..G..S..C..L..E..F..L..S..W..L..P..S..V..M..D..C..D..L..G..V..A..N..K..L..I..K..E..E..L..Q

**M** I..A..N..N..G..S..S..Q------------------------------------------------------------------------..A..N..K..L..I..K..E..E..L..Q

**R** I..A..N..N..G..S..S..Q------------------------------------------------------------------------..A..N..K..L..I..K..E..E..L..Q

**H** V..A..N..N..G..S..S..Q------------------------------------------------------------------------..A..N..K..L..I..K..E..E..L..Q

*LIG1 (isotig22978)*

**S** accccaagggaaaacctcacagaggccaaagaggtaaaacagaaggacgatgagggcgaccaagccacaccaggctctgagcctacaacaccacctaagacccca

**M** accccaaaagaaagcctcgcagaggctgaagacgt----------------------------------------------------------------------

**R** accccaacagaaagcctcacagaggctgaagaagt----------------------------------------------------------------------

**H** accccgaaagaaagcctcacagaggctgaagtggc----------------------------------------------------------------------

***** * **** **** ******* *** *

**S** ..T..P..R..E..N..L..T..E..A..K..E..V..K..Q..K..D..D..E..G..D..Q..A..T..P..G..S..E..P..T..T..P..P..K..T..P

**M** ..T..P..K..E..S..L..A..E..A..E..D..----------------------------------------------------------------------

**R** ..T..P..T..E..S..L..T..E..A..E..E..----------------------------------------------------------------------

**H** ..T..P..K..E..S..L..T..E..A..E..V..----------------------------------------------------------------------

**S** aagacccccattcggaagcagctcctgaaacggatgaatcgggatgttctggaagagcagaaagaggaaaaggacagaggaaccaagaaaaggaagaaggaagaa

**M** ---------------------------------------------------------------------------------------------------------

**R** ---------------------------------------------------------------------------------------------------------

**H** ---------------------------------------------------------------------------------------------------------

**S** ..K..T..P..I..R..K..Q..L..L..K..R..M..N..R..D..V..L..E..E..Q..K..E..E..K..D..R..G..T..K..K..R..K..K..E..E

**M** ---------------------------------------------------------------------------------------------------------

**R** ---------------------------------------------------------------------------------------------------------

**H** ---------------------------------------------------------------------------------------------------------

**S** gcagagattccaagccttacagagaccaaagaggcaaaacagaaggaagaagaggagggggacaagcccacgacaccacccgagaccctaaagaccccc

**M** -----------------------------------aaaacagaaggaagaaaaggagggggaccagctcatagtcccctctgagcccacaaagtcccct

**R** -----------------------------------aaaacagaaggaagaacaggtggaggaccagcccacagtaccccctgagcccacagagtcccct

**H** -----------------------------------cacagagaaggaaggagaagacggggaccagcccaccacgcctcccaagcccctaaagacctcc

* * ********* * * * * **** *** ** ** * ** ** * ** ** *

**S** ..A..E..I..P..S..L..T..E..T..K..E..A..K..Q..K..E..E..E..E..G..D..K..P..T..T..P..P..E..T..L..K..T..P

**M** -----------------------------------V..K..Q..K..E..E..K..E..G..D..Q..L..I..V..P..S..E..P..T..K..S..P

**R** -----------------------------------V..K..Q..K..E..E..Q..V..E..D..Q..P..T..V..P..P..E..P..T..E..S..P

**H** -----------------------------------A..T..E..K..E..G..E..D..G..D..Q..P..T..T..P..P..K..P..L..K..T..S

*Ercc5 (isotig14243)*

**S** aggtgtgtgctgggggtgatgtgccagagaaagggacagcattgagaactccaggcagtgaagaagatgggaaagtgtatgctggtggtgataggtctggagaaggaccagcgatgagag

**M** aagtgtctggcagcagtgat----------------------------------------------------------------------------------------------------

**R** gaatgtgtgccagcagtgat----------------------------------------------------------------------------------------------------

**H** aagtgtgtgctggggatgatgtgcagacggga----------------------------------------------------------------------------------------

*** ** * ****

**S** .K..V..C..A..G..G..D..V..P..E..K..G..T..A..L..R..T..P..G..S..E..E..D..G..K..V..Y..A..G..G..D..R..S..G..E..G..P..A..M..R.

**M** .K..V..S..G..S..S..D----------------------------------------------------------------------------------------------------

**R** .R..M..C..A..S..S..D----------------------------------------------------------------------------------------------------

**H** .K..V..C..A..G..D..D..V..Q..T..G----------------------------------------------------------------------------------------

**S** cactgggtgatgccatagatgggaacgtgtgtgctggtgctaataagcctgtggaggggccagtgctgagagtactgcttgga

**M** --------------------------------------------------------gatctagcagagaaaatgctgcttgga

**R** --------------------------------------------------------aatctggccgtggaaatgctgcttgga

**H** --------------------------------------------------------gggccaggagcagaagaaatgcgtata

* * * *** * *

**S** .A..L..G..D..A..I..D..G..N..V..C..A..G..A..N..K..P..V..E..G..P..V..L..R..V..L..L..G

**M** --------------------------------------------------------..D..L..A..E..K..M..L..L..G

**R** --------------------------------------------------------..N..L..A..V..E..M..L..L..G

**H** --------------------------------------------------------..G..P..G..A..E..E..M..R..I

*Rtn3 (mid1.contig* *23036)*

**S** tccacggaagaaaccagtggcagagatgtgcaaggcagtgcacagatacagggtaatgtgctttcagagctgcccacagctccaggtggtaatgtgctttcagagctgccca

**M** tccacaaaagaagctggtggcaacggtgtgccaggcagttctcag-------------------------------------------------------------------

**R** tccacaaaagaagctggtggcaaaggtgtaccagacagttctcag-------------------------------------------------------------------

**H** tccacaaaagaattcagtatcaaaggtgtgcaaggcaatatgcagaaacag-------------------------------------------------------------

***** ***** ** ** * *** * ** ** * ***

**S** ..S..T..E..E..T..S..G..R..D..V..Q..G..S..A..Q..I..Q..**G..N..V..L..S..E..L..P..T..A..P..G**..**G..N..V..L..S..E..L..P**.

**M** ..S..T..K..E..A..G..G..N..G..V..P..G..S..S..Q-------------------------------------------------------------------

**R** ..S..T..K..E..A..G..G..K..G..V..P..D..S..S..Q-------------------------------------------------------------------

**H** ..S..T..K..E..F..S..I..K..G..V..Q..G..N..M..Q..K..Q-------------------------------------------------------------

**S** cagctccaggtggtaatgtgctttcagagctgcccacagctccaggtgggaaacccatcgccctgagttccggagcagccactgtg

**M** -----------------cttcattctgagctgcctggctctatgcctgagaaatgggtctc---aggctctggagcagccacagtg

**R** -----------------------------------------------------cctatctc---aggctctggagcagccacagtg

**H** -----------gatgacacacttgcagaattacctggatctccacctgagaaatgtgactctttgggttctggagtggccacagtg

* * * ** **** ***** ***

**S** .**T..A..P..G**..G..N..V..L..S..E..L..P..T..A..P..G..G..K..P..I..A..L..S..S..G..A..A..T..V

**M** -----------------..L..H..S..E..L..P..G..S..M..P..E..K..W..V..---S..G..S..G..A..A..T..V

**R** -----------------------------------------------------..P..I..---S..G..S..G..A..A..T..V

**H** -----------..D..D..T..L..A..E..L..P..G..S..P..P..E..K..C..D..S..L..G..S..G..V..A..T..V

*CINAP(isotig22590)*

**S** acaataagatcactaacaactatgagagcgctgctgctgctgctgctgctgctgctgctgctgctgctgctgctgctgctgctcagagtctagacaacagtgaaaacccagaa

**M** acaccaagatcactgacaacaaagggagcgttgccgcaaatccagat---------------------------------------------gacaacagtgatgacccagag

**R** ac---aagatcactgacaacaaagggagcattgccaccggtccagat---------------------------------------------gacaacggtgacaagccagag

**H** acaacgagaccactgataacaacgagagtgctgatgaccacgaaaccactgacaacaatgagagtgcagat---------------------gacaacaacgagaatcctgaa

** *** **** * *** * * *** ** ****** ** * ** **

**S** .N..N..K..I..T..N..N..Y..E..S..A..A..A..A..A..A..A..A..A..A..A..A..A..A..A..A..A..A..Q..S..L..D..N..S..E..N..P..E

**M** .N..T..K..I..T..D..N..K..G..S..V..A..A..N..P..D---------------------------------------------..D..N..S..D..D..P..E

**R** .H---..K..I..T..D..N..K..G..S..I..A..T..G..P..D---------------------------------------------..D..N..G..D..K..P..E

**H** .N..N..E..T..T..D..N..N..E..S..A..D..D..H..E..T..T..D..N..N..E..S..A..D---------------------..D..N..N..E..N..P..E

*Sarcalumenin (isotig12112)*

**S** gcagcctctgacactgggtctcctggagtagaggtagaggaagaaggtgaggaaggggcagtagaccaactccaggtccgtgcctctcccacagatggtgaggccagccccggaccggat

**M** gcagcctctgacacggcatcttctggagtagaggcagaagaagg----------------------------------------------------------------------------

**R** gcagtctctgacatggcatcttccgaagtaggggcagaggaaga----------------------------------------------------------------------------

**H** ------------------------------------------------------------------------------------------------------------------------

**S** ..A..A..S..D..T..G..S..P..G..V..E..V..E..E..E..G..E..E..G..A..V..D..Q..L..Q..V..R..A..S..P..T..D..G..E..A..S..P..G..P..D

**M** ..A..A..S..D..T..A..S..S..G..V..E..A..E..E..----------------------------------------------------------------------------

**R** ..A..V..S..D..M..A..S..S..E..V..G..A..E..E..----------------------------------------------------------------------------

**H** ------------------------------------------------------------------------------------------------------------------------

**S** cgccagtccacagagctagatacagctgctgatgccatatctgctggagaagaatcaaaggctgag

**M** --------------------------------------------tgcagaggaatcaaaggctgag

**R** --------------------------------------------cgctgaagactcaaaggctgag

**H** --------------------------------------------tgggggagagtcggagccagat

* * ** ** ** * **

**S** ..R..Q..S..T..E..L..D..T..A..A..D..A..I..S..A..G..E..E..S..K..A..E

**M** --------------------------------------------G..A..E..E..S..K..A..E

**R** --------------------------------------------D..A..E..D..S..K..A..E

**H** --------------------------------------------V..G..G..E..S..E..P..D

*Sec23a (isotig20294)*

**S** tgtcctaatctcactggcagggtctcccagctggactggaattcactacatggaccaggctggcctcgatctcacaaagatccatctctgtcttctcagtgttgggatcaaagaggatacatggtaatgggtgc

**M** tgtcctaaccttactg------------------------------------------------------------------------------------------------gaggatacatggtaatgggtgac

**R** tgccctaaccttactg------------------------------------------------------------------------------------------------gaggatacatggtaatgggtgac

**H** tgtcccaaccttactg------------------------------------------------------------------------------------------------gaggatacatggtaatgggtgat

** ** ** ** **** **********************

**S** ..C..P..N..L..T..G..R..V..S..Q..L..D..W..N..S..L..H..G..P..G..W..P..R..S..H..K..D..P..S..L..S..S..Q..C..W..D..Q..R..G..Y..M..V..M..G..D

**M** ..C..P..N..L..T.------------------------------------------------------------------------------------------------.G..G..Y..M..V..M..G..D

**R** ..C..P..N..L..T.------------------------------------------------------------------------------------------------.G..G..Y..M..V..M..G..D

**H** ..C..P..N..L..T.------------------------------------------------------------------------------------------------.G..G..Y..M..V..M..G..D

*Rpl4(isotig04552)*

**S** aaactggaagcagtagcagcagcactggcagccaaatcagagaagggggcagcagagaagagggctgcagcccctgcagagaaaggagcagcagagaagagggctgcagcccctgcagagaaaggagcagcagag

**M** aagctggaagcagcagctactgcactggcaaccaaatccgagaag------------------------------------------------------------------------------------------

**R** aagctggaagccgcagctgctgcactggcagccaaatcggagaag------------------------------------------------------------------------------------------

**H** aaggcagctgctgcagcagcggcactacaagccaaatcagatgag------------------------------------------------------------------------------------------

** * ** * *** * ***** * ******* ** **

**S** ..K..L..E..A..V..A..A..A..L..A..A..K..S..E..K..G..A..A..E..K..R..A..A..A..P..A..E..K..G..A..A..E..K..R..A..A..A..P..A..E..K..G..A..A..E

**M** ..K..L..E..A..A..A..T..A..L..A..T..K..S..E..K------------------------------------------------------------------------------------------

**R** ..K..L..E..A..A..A..A..A..L..A..A..K..S..E..K------------------------------------------------------------------------------------------

**H** ..K..A..A..A..A..A..A..A..L..Q..A..K..S..D..E------------------------------------------------------------------------------------------

**S** aagagggctgcagcccctgcagagaagggggctgcaagcaagaagcctatggagaagaagaaa

**M** ------------gttgttccagagaaggggactgcagacaagaagccagcggtaggcaagaaa

**R** ------------attgttccagagaagggggctggagacaaaaaacctgcagtaggcaaaaaa

**H** ---------------------aaggcggcggttgcaggcaagaagcctgtggtaggtaagaaa

** ** * ** * *** ** ** * ** ***

**S** ..K..R..A..A..A..P..A..E..K..G..A..A..S..K..K..P..M..E..K..K..K

**M** ------------..V..V..P..E..K..G..T..A..D..K..K..P..A..V..G..K..K

**R** ------------..I..V..P..E..K..G..A..G..D..K..K..P..A..V..G..K..K

**H** ---------------------..K..A..A..V..A..G..K..K..P..V..V..G..K..K

*Parp9 (isotig17313)*

**S** caggatcacgcatggtcacaggagcatgcatggtcacaggctcacacccagtcacaggatcatgcatggtcacaggatcgcacatggccacacaattatttgtcaggacca---atgttttcttcacagcaatcc

**M** caggataggacattctcacagcatccgatgtggtcacagg------------------------------------------------------gctactcatcaggaccaggaatggtctcttcgctgcagtcc

**R** caggataggacattctcacagcatccaatgtggtcccagg------------------------------------------------------gctactcgtcagaaccaggaatggcctcttcgctgcagtcc

**H** caggaatatgtacagtcacaag------------------------------------------------------------------------attactcatcaggaccaatgagaccctttgcacagcatcct

***** * ***** ** * **** **** * * * * * *** *

**S** ..Q..D..H..A..W..S..Q..E..H..A..W..S..Q..A..H..T..Q..S..Q..D..H..A..W..S..Q..D..R..T..W..P..H..N..Y..L..S..G..P---..M..F..S..S..Q..Q..S

**M** ..Q..D..R..T..F..S..Q..H..P..M..W..S..Q.------------------------------------------------------.G..Y..S..S..G..P..G..M..V..S..S..L..Q..S

**R** ..Q..D..R..T..F..S..Q..H..P..M..W..S..Q.------------------------------------------------------.G..Y..S..S..E..P..G..M..A..S..S..L..Q..S

**H** ..Q..E..Y..V..Q..S..Q.------------------------------------------------------------------------.D..Y..S..S..G..P..M..R..P..F..A..Q..H..P

*Mtap7d2(isotig29476)*

**S** aagcacgcggctgacaagcgggccactgagaagcctgtggcagacaagcataccactgagaagtacgcagctgacaaacatgccgttgagaagtacttggctgacaagcatgctactgagaagcatgctgccaca

**M** aagcgcatggctgacaag------------------------------------------------------------tatgccaccgagaagtatgtagcagataagcatgcaactgagaaacattctgcccct

**R** aagcccatggctgacaag------------------------------------------------------------gatgccactgagaagtatgtagctgataagcatgcaactgagaaacattctgccacc

**H** aagcatgtagtggacaag------------------------------------------------------------catgccagcgagaagcatgc---------------------------tgctgccgca

**** * ****** ***** ****** * * ***** *

**S** ..K..H..A..A..D..K..R..A..T..E..K..P..V..A..D..K..H..T..T..E..K..Y..A..A..D..K..H..A..V..E..K..Y..L..A..D..K..H..A..T..E..K..H..A..A..T

**M** ..K..R..M..A..D..K------------------------------------------------------------..Y..A..T..E..K..Y..V..A..D..K..H..A..T..E..K..H..S..A..P

**R** ..K..P..M..A..D..K------------------------------------------------------------..D..A..T..E..K..Y..V..A..D..K..H..A..T..E..K..H..S..A..T

**H** ..K..H..V..V..D..K------------------------------------------------------------..H..A..S..E..K..H..---------------------------A..A..A..A

*Melusin (isotig19920)*

**S** ctccgagaaaggcctaagagtagagcatgtaccacctgtggaggtcagagcacgatttacaagactccttccaccatgtgggtcctggggattgcattcatcaggcttggtggcagatcagagttgcctccaaaa

**M** ttccgagaaaggcctaag---------------------------------------------------------------------------------------------------tctgagatgcctcccaaa

**R** ctccgagaaaggcctaag---------------------------------------------------------------------------------------------------tctgagatgcctccgaaa

**H** cgccgggagaggcccaag---------------------------------------------------------------------------------------------------tcagagttgcctctgaag

*** ** ***** *** ** *** ****** **

**S** ..L..R..E..R..P..K..S..R..A..C..T..T..C..G..G..Q..S..T..I..Y..K..T..P..S..T..M..W..V..L..G..I..A..F..I..R..L..G..G..R..S..E..L..P..P..K

**M** ..F..R..E..R..P..K---------------------------------------------------------------------------------------------------..S..E..M..P..P..K

**R** ..L..R..E..R..P..K---------------------------------------------------------------------------------------------------..S..E..M..P..P..K

**H** ..R..R..E..R..P..K---------------------------------------------------------------------------------------------------..S..E..L..P..L..K

*Ifi204(isotig12974)*

**S** agcaatttctttgccaagaaacagaagagctctatccaaaacttcggtgacataaatggaaacatgctctcccagctgagccaatttgcaacaacttcagctatcagcatagattttgctgagagtcaagttcag

**M** aacattccttcggctaagaaccaaaaat-----------------------------------------------------------------------------------------------------------

**R** aactctcctttggctaagaaccaaaata-----------------------------------------------------------------------------------------------------------

**H** aacacttcgtttactccgaatcaggaaa-----------------------------------------------------------------------------------------------------------

* * * * * *** ** *

**S** ..S..N..F..F..A..K..K..Q..K..S..S..I..Q..N..F..G..D..I..N..G..N..M..L..S..Q..L..S..Q..F..A..T..T..S..A..I..S..I..D..F..A..E..S..Q..V..Q

**M** ..N..I..P..S..A..K..N..Q..K.-----------------------------------------------------------------------------------------------------------

**R** ..N..S..P..L..A..K..N..Q..N.-----------------------------------------------------------------------------------------------------------

**H** ..N..T..S..F..T..P..N..Q..E.-----------------------------------------------------------------------------------------------------------

**S** actcctcccaaacttcctccaaatgcttccaggcattcttcatcaaagtctcctcaggttcctccagcaacactgtccctgggtatccaggtcggacaggtgcctccagtgaaagcatccagtattctcccggct

**M** ---------------------------------------------------------------------------------------------------------------------------------------

**R** ---------------------------------------------------------------------------------------------------------------------------------------

**H** ---------------------------------------------------------------------------------------------------------------------------------------

**S** ..T..P..P..K..L..P..P..N..A..S..R..H..S..S..S..K..S..P..Q..V..P..P..A..T..L..S..L..G..I..Q..V..G..Q..V..P..P..V..K..A..S..S..I..L..P..A

**M** ---------------------------------------------------------------------------------------------------------------------------------------

**R** ---------------------------------------------------------------------------------------------------------------------------------------

**H** ---------------------------------------------------------------------------------------------------------------------------------------

**S** cctcctgtgcctccatcaacaacttcatggctgtttccaacagtatccaatgggctccaaacttttcaaagaattccatgtgcagtacccagcaacttgtctcctttggtgactacagtcacgtcactcatgaat

**M** ---------------------------------------------------------------------------------------------------------------------------------------

**R** ---------------------------------------------------------------------------------------------------------------------------------------

**H** ---------------------------------------------------------------------------------------------------------------------------------------

**S** ..P..P..V..P..P..S..T..T..S..W..L..F..P..T..V..S..N..G..L..Q..T..F..Q..R..I..P..C..A..V..P..S..N..L..S..P..L..V..T..T..V..T..S..L..M..N

**M** ---------------------------------------------------------------------------------------------------------------------------------------

**R** ---------------------------------------------------------------------------------------------------------------------------------------

**H** ---------------------------------------------------------------------------------------------------------------------------------------

**S** cagaggccaccggtccaaactcaagtgggtaccaga

**M** -------cacaaccccagaatcagaacattcccaga

**R** -------tacagacccaaaaccagaacattcccaga

**H** -------cccaggcccaacggcaggtggatgcaaga

* *** ** * * ***

**S** ..Q..R..P..P..V..Q..T..Q..V..G..T..R

**M** -------.S..Q..P..Q..N..Q..N..I..P..R

**R** -------.I..Q..T..Q..N..Q..N..I..P..R

**H** -------.T..Q..A..Q..R..Q..V..D..A..R

*Ift7ip (isotig16621)*

**S** acctccgatgattcaccttcagaacagctgccttctgctgctcagtcttctggagagcctgccagtagtggactggctcctgataatctggtctctgatgatggg---------------gcttctggtgaactg

**M** ccctccggtgattcacagtc------------------------------------------------------------tgatgagccaccttctagtgaggactcccttcctaggtctgtctgcagcggactg

**R** acctctggtgactcgccttc------------------------------------------------------------tgatgatctgccttctagtgaggagtcgcttcgtacgtctgtctgcagcagactg

**H** gcctctgatgatctggcctc------------------------------------------------------------tggtgatctatcctctagtgaactg---------------gcctctgatgatctg

**** * *** ** ** * * * *** *** * * ***

**S** ..T..S..D..D..S..P..S..E..Q..L..P..S..A..A..Q..S..S..G..E..P..A..S..S..G..L..A..P..D..N..L..V..S..D..D..G---------------..A..S..G..E..L

**M** ..P..S..G..D..S..Q..------------------------------------------------------------S..D..E..P..P..S..S..E..D..S..L..P..R..S..V..C..S..G..L

**R** ..T..S..G..D..S..P..------------------------------------------------------------S..D..D..L..P..S..S..E..E..S..L..R..T..S..V..C..S..R..L

**H** ..A..S..D..D..L..A..------------------------------------------------------------S..G..D..L..S..S..S..E..L---------------..A..S..D..D..L

*Serping1 (isotig42961)*

**S** tccttcacccagtacacccaaccagcctcccagccccctacagattctcccagccagcccccaacagacatttctagccagccctctacaaccacagattctcctagtcagcctcccacagattcttccagccaa

**M** tccttcagccagcacagccagccagctgctcagc------------------------------------------------------tacccacagattctccaggacagccccctctgaattcttccagccag

**R** tccttcatccagcatgtccaaccagctgctcagc------------------------------------------------------tacctgaggattctcccagccagtctcccgtgaattcttccagcccg

**H** cccacca-----cagagcccaccacccaacccac------------------------------------------------------catcc---------------------------aacccacccaaccaa

** ** * ** *** * * * * * * * *** **

**S** ..S..F..T..Q..Y..T..Q..P..A..S..Q..P..P..T..D..S..P..S..Q..P..P..T..D..I..S..S..Q..P..S..T..T..T..D..S..P..S..Q..P..P..T..D..S..S..S..Q

**M** ..S..F..S..Q..H..S..Q..P..A..A..Q.------------------------------------------------------.L..P..T..D..S..P..G..Q..P..P..L..N..S..S..S..Q

**R** ..S..F..I..Q..H..V..Q..P..A..A..Q.------------------------------------------------------.L..P..E..D..S..P..S..Q..S..P..V..N..S..S..S..P

**H** ..P..T.-----.T..E..P..T..T..Q..P..------------------------------------------------------T..I.---------------------------.Q..P..T..Q..P.
