# Supplementary material for: Transcriptome Sequencing of the Blind Subterranean Mole Rat, Spalax galili: Utility and Potential for the Discovery of Novel Evolutionary Patterns
Source: PLoS One. 2011 Aug 12;6(8):e21227. doi: 10.1371/journal.pone.0021227 (PMC3155515; doi:10.1371/journal.pone.0021227)
Supplement: Table S2 — Top 50 genes with the most significant differences in read counts for Spalax muscle/hypoxia vs. muscle/normoxia. The titles ‘#n’, and ‘#h’, denote: number of reads in muscle/normoxia vs. muscle/hypoxia libraries. Note that the total number of reads assembled from muscle/hypoxia (C.4) is about 2.4 times larger than those from muscle/normoxia (C.3). The conducted differential expression test (see Methods) takes into account the global difference in read counts between different tissues/treatments. Gene names in black bold fonts designate genes that were previously identified as differentially expressed by microarray data. Gene names in gray bold fonts indicate that differentially expressed isozymes or paralogs were found previously. FDR column denotes adjusted P values. (DOC) [file pone.0021227.s006.doc]

**Table S2.**

| **Isotig name** | **#n** | **#h** | **FDR** | **gene name** |
| --- | --- | --- | --- | --- |
| isotig03675 | 144 | 1926 | 3.1E-183 | lipin 1 |
| isotig22428 | 159 | 1847 | 7.0E-159 | xin actin-binding repeat containing 1 |
| isotig16056 | 31 | 693 | 1.8E-85 | ERBB receptor feedback inhibitor 1 |
| isotig03993 | 29 | 554 | 1.2E-63 | G protein-coupled receptor 157 |
| isotig05949 | 185 | 1273 | 8.5E-63 | **glutamate-ammonia ligase (glutamine synthetase)** |
| isotig32854 | 19 | 391 | 2.6E-46 | OTU domain containing 1 |
| isotig05974 | 42 | 518 | 5.4E-46 | syndecan 4 |
| contig57880 | 3872 | 11750 | 6.1E-43 | titin |
| isotig23386 | 5 | 273 | 5.2E-42 | similar to hypothetical protein MGC6835 |
| isotig01962 | 37 | 454 | 4.0E-40 | **ankyrin repeat domain 1 (cardiac muscle)** |
| isotig01944 | 167 | 952 | 1.7E-34 | **splicing factor, arginine/serine-rich 3** |
| isotig33484 | 37 | 417 | 1.9E-34 | leucine rich repeat containing 30 |
| isotig08700 | 28 | 356 | 3.4E-32 | FK506 binding protein 5 |
| isotig19475 | 10 | 249 | 1.0E-31 | **forkhead box O1** |
| isotig18032 | 16 | 282 | 5.4E-31 | **activating transcription factor 3** |
| isotig06808 | 23 | 321 | 6.4E-31 | 6-phosphofructo-2-kinase/fructose-2,6-biphosphatase 3 |
| contig11513 | 12 | 250 | 1.1E-29 | elongation factor RNA polymerase II 2 |
| isotig14054 | 40 | 394 | 5.1E-29 | **regulator of calcineurin 1** |
| isotig00986 | 81 | 567 | 1.9E-28 | ubiquitin interaction motif containing 1 |
| isotig13244 | 59 | 464 | 5.6E-27 | nuclear receptor interacting protein 1 |
| contig35926 | 22 | 286 | 3.1E-26 | stomatin; ABO-family member 5 |
| isotig41385 | 39 | 368 | 5.3E-26 | **jumonji domain containing 1C** |
| isotig11426 | 30 | 323 | 1.1E-25 | bestrophin 3 |
| isotig09145 | 51 | 415 | 4.1E-25 | enhancer trap locus 4 |
| isotig08325 | 14 | 234 | 5.1E-25 | GRAM domain containing 1B |
| isotig00525 | 47 | 388 | 7.1E-24 | GABA(A) receptor-associated protein like 1 |
| contig01113 | 732 | 2579 | 2.7E-23 | ENSRNOG00000022637 |
| isotig07502 | 26 | 284 | 7.0E-23 | E2F transcription factor 8 |
| isotig33664 | 83 | 523 | 1.8E-22 | zinc finger and BTB domain containing 16 |
| isotig39738 | 16 | 211 | 1.8E-19 | DNA-damage-inducible transcript 4 |
| isotig05647 | 204 | 913 | 2.9E-19 | zinc finger, AN1-type domain 5 |
| isotig21916 | 19 | 222 | 9.0E-19 | kelch-like 25 (Drosophila) |
| isotig21658 | 27 | 258 | 2.3E-18 | Rap guanine nucleotide exchange factor (GEF) 2 |
| isotig01003 | 64 | 388 | 1.6E-15 | **cysteine and glycine-rich protein 3** |
| isotig41520 | 74 | 424 | 2.3E-15 | sterile alpha motif domain containing 8 |
| isotig15253 | 63 | 373 | 2.6E-14 | actin-binding Rho activating protein |
| isotig05104 | 16 | 177 | 2.9E-14 | **cyclin L1** |
| isotig03999 | 151 | 675 | 4.2E-14 | apoptosis-inducing factor, mitochondrion-associated 2 |
| isotig41645 | 19 | 190 | 4.8E-14 | **TCDD-inducible poly(ADP-ribose) polymerase** |
| isotig20970 | 71 | 398 | 7.6E-14 | HMG-box transcription factor 1 |
| contig41103 | 92 | 472 | 9.2E-14 | solute carrier family 25 |
| isotig40667 | 5 | 113 | 9.6E-14 | connective tissue growth factor |
| isotig04254 | 236 | 935 | 1.8E-13 | DIP2 disco-interacting protein 2 homolog A |
| contig33079 | 121 | 558 | 1.2E-12 | **pyruvate dehydrogenase kinase, isozyme 4** |
| isotig04715 | 65 | 364 | 1.3E-12 | myosin binding protein H |
| isotig00769 | 475 | 1625 | 1.6E-12 | **filamin C, gamma (actin binding protein 280)** |
| isotig21301 | 79 | 410 | 3.1E-12 | **protein phosphatase 2 (formerly 2A), catalytic subunit, alpha isoform** |
| isotig04028 | 119 | 544 | 4.7E-12 | leucine-rich repeats and immunoglobulin-like domains 1 |
| isotig16876 | 16 | 161 | 6.0E-12 | ENSRNOG00000007426 |
| isotig27501 | 13 | 145 | 9.7E-12 | chemokine (C-X-C motif) receptor 7 |
