# Supplementary material for: Transcriptome Sequencing of the Blind Subterranean Mole Rat, Spalax galili: Utility and Potential for the Discovery of Novel Evolutionary Patterns
Source: PLoS One. 2011 Aug 12;6(8):e21227. doi: 10.1371/journal.pone.0021227 (PMC3155515; doi:10.1371/journal.pone.0021227)
Supplement: Table S3 — The titles ‘#n’, and ‘#h’, denote: number of reads in brain/normoxia vs. brain/hypoxia libraries. Note that the total number of reads assembled from brain/hypoxia (C.2) is about 1.8 times larger than those from brain/normoxia (C.1). The conducted differential expression test (see Methods) takes into account the global difference in read counts between different tissues/treatments. (DOC) [file pone.0021227.s007.doc]

**Table S3.**

| **isotigs** | **#n** | **#h** | **FDR** | **Gene Name** |
| --- | --- | --- | --- | --- |
| isotig08143 | 46 | 417 | 2.5E-39 | actinin alpha 4 |
| isotig20973 | 11 | 153 | 5.9E-18 | transmembrane protein 208 |
| isotig05290 | 45 | 254 | 2.2E-14 | cytochrome c oxidase subunit IV isoform 1 |
| isotig08761 | 1 | 77 | 9.9E-13 | cytochrome P450, family 4, subfamily b, polypeptide 1 |
| isotig47698 | 42 | 224 | 9.4E-12 | solute carrier family 23 (nucleobase transporters), member 2 |
| isotig14978 | 21 | 138 | 5E-09 | CDKN2A interacting protein N-terminal like |
| isotig17373 | 3 | 60 | 1.2E-07 | myc induced nuclear antigen; similar to myc induced nuclear antigen |
| contig61357 | 23 | 134 | 1.5E-07 | phosphofructokinase, muscle |
| isotig24305 | 13 | 92 | 3.2E-06 | guanine nucleotide binding protein (G protein), beta polypeptide 2 |
| isotig17372 | 6 | 62 | 1.5E-05 | beta-gamma crystallin domain containing 3 |
| isotig42407 | 55 | 207 | 1.8E-05 | phosphatidylinositol-specific phospholipase C, X domain containing 2; CD96 molecule |
| isotig41125 | 59 | 214 | 3.5E-05 | eukaryotic translation elongation factor 1 alpha 1-like |
| contig00394 | 47 | 181 | 5.5E-05 | DEAD (Asp-Glu-Ala-Asp) box polypeptide 3, X-linked |
| isotig31682 | 35 | 146 | 0.00011 | prostaglandin D2 synthase (brain) |
| isotig22241 | 39 | 154 | 0.0002 | WW domain binding protein 2 |
| isotig25502 | 5 | 49 | 0.0004 | TIP41, TOR signaling pathway regulator-like (S. cerevisiae) |
| isotig43077 | 6 | 52 | 0.00055 | neuroligin 2 |
| isotig39702 | 24 | 107 | 0.00085 | FK506 binding protein 9, 63 kDa |
| isotig08700 | 4 | 41 | 0.00155 | FK506 binding protein 5 |
| isotig08916 | 23 | 101 | 0.00169 | ribosomal protein L10; |
| isotig05974 | 22 | 96 | 0.00285 | syndecan 4 |
| isotig06808 | 20 | 90 | 0.00301 | 6-phosphofructo-2-kinase/fructose-2,6-biphosphatase 3 |
| isotig05919 | 11 | 63 | 0.00326 | nucleolar complex associated 2 homolog (S. cerevisiae) |
| isotig21642 | 8 | 53 | 0.00335 | ADAM metallopeptidase with thrombospondin type 1 motif, 1 |
| isotig22743 | 34 | 126 | 0.00385 | hemoglobin, beta |
| isotig07905 | 3 | 34 | 0.00394 | U2 small nuclear RNA auxiliary factor 1-like 4 |
| isotig21903 | 13 | 68 | 0.00406 | similar to 40S ribosomal protein S2; ribosomal protein S2 |
| isotig40387 | 17 | 76 | 0.01092 | nephroblastoma overexpressed gene |
| isotig42916 | 8 | 49 | 0.01096 | leucine rich repeat containing 8 family, member A |
| isotig37166 | 4 | 35 | 0.01115 | coiled-coil domain containing 25 |
| isotig08965 | 15 | 70 | 0.01122 | ENSRNOG00000008877 |
| isotig40410 | 13 | 64 | 0.01174 | early endosome antigen 1 |
| isotig11185 | 18 | 78 | 0.01251 | RGD1563224 |
| isotig21193 | 15 | 69 | 0.01423 | RIO kinase 3 (yeast) |
| isotig19499 | 8 | 47 | 0.01785 | endothelin converting enzyme 1 |
| isotig17299 | 9 | 50 | 0.0184 | protein phosphatase 1, regulatory (inhibitor) subunit 3C |
| isotig47679 | 24 | 92 | 0.01843 | polo-like kinase 2 (Drosophila) |
| isotig08113 | 18 | 76 | 0.01848 | phospholipid transfer protein |
| isotig15188 | 2 | 25 | 0.01969 | similar to KIAA0892 protein |
| isotig35150 | 12 | 58 | 0.02321 | ENSRNOG00000005884 |
| isotig17227 | 0 | 17 | 0.02472 | ENSRNOG00000005608 |
| isotig09650 | 0 | 17 | 0.02472 | tubulin, gamma 2; similar to tubulin, gamma 2 |
| isotig07720 | 4 | 32 | 0.02515 | phosphomannomutase 2 |
| isotig36818 | 4 | 32 | 0.02515 | K(lysine) acetyltransferase 5 |
| isotig19277 | 1 | 19 | 0.02963 | AE binding protein 2 |
| isotig20735 | 15 | 65 | 0.0297 | TBC1 domain family, member 14 |
| isotig15637 | 8 | 44 | 0.03372 | programmed cell death 10 |
| isotig40729 | 0 | 16 | 0.03412 | RCSD domain containing 1 |
| isotig41927 | 0 | 16 | 0.03412 | pro-melanin-concentrating hormone |
| isotig23386 | 0 | 16 | 0.03412 | similar to hypothetical protein MGC6835 |
