# Supplementary material for: Transcriptome Sequencing of the Blind Subterranean Mole Rat, Spalax galili: Utility and Potential for the Discovery of Novel Evolutionary Patterns
Source: PLoS One. 2011 Aug 12;6(8):e21227. doi: 10.1371/journal.pone.0021227 (PMC3155515; doi:10.1371/journal.pone.0021227)
Supplement: Table S4 — Examples of non-conserved/weakly-conserved S. galili transcribed regions. Column ‘Repeat’: Repeat Masker output for the non-conserved Spalax region; Columns ‘Spalax’/’rat’: electrophoresis and sequencing results of RT-PTR products (where ‘√’ and ‘×’ indicate that the expected band was detected/not detected in cDNAs, blank cells denote untested cases; ORF/PTC+ indicate whether the tested novel transcribed regions are part of an ORF or contain a termination codon). ‘*’ indicates that though the tested transcribed region was not found experimentally in rat it shows weak similarity to introns of some target species, but not to known exons. Size: denotes novel region size in bp. (DOC) [file pone.0021227.s008.doc]

**Table S4.**

| **ID** | **Gene** | **Repeat** | ***Spalax*** | **Rat** | **Region** | **Size** | **Comments** |
| --- | --- | --- | --- | --- | --- | --- | --- |
| isotig22590 | *Cinap* | Simple repeat | √ ORF | × | insertion | 30-60 | Non-conserved highly polymorphic poly-Alanine (10-20 repeats) in *Cinap* gene. *Tbr1-Cask-Cinap* complex is involved regulation of NMDA receptor subunit NR2b in hippocampal neurons [1,2] |
| C1.contig23036 | *Rtn3* | aa repeat | √ ORF | × | insertion | 75 | (GNVLSELPTAPG) repeat, which seems to be polymorphic. *Reticulon-3* gene is involved in different developmental processes, including brain development. |
| isotig19920 | *melusin* | *SINE/B4* | √ ORF | × | alt.splice | 99 | Genes essential in preserving cardiac function in response to pressure overload [3,4,5,6] |
| C4.contig27611 | *Nup85* | *SINE/Alu* | √ ORF | ×* | alt.splice | 90 |
| isotig12112 | *Srl* |  | √ ORF | × | insertion | 120 |
| isotig20772 | *TppII* | *LTR/ERVL-MaLR* | √ ORF | × | alt.splice | 72 | Multi-functional genes known to be involved in the response to DNA damage and repair [7,8,9] |
| isotig22978 | *ligase I* |  | √ ORF | × | insertion | 111 |
| isotig14243 | *Ercc5* |  | √ ORF | × | alt.splice | 156 |
| isotig20294 | *Sec23A* | *SINE/Alu,* | √ ORF | ×* | alt.splice | 96 | Region proximal to a conserved phenylalanine residue which, when mutated to Cysteine, prevents normal skull and face development in human [10] |
| isotig00412 | *Ncb5or* | *SINE/Alu* | √ PTC+ | × | alt.splice | 102 | Strongly translated PTC+ *Spalax* transcript of the redox gene *Ncb5or* which is involved in endolasmic reticulum stress response. |
| isotig12974 | *Ifi204* |  | ORF |  | insertions | >300 | *Ifi204* may regulate the balance between proliferation and differentiation [11]. Iﬁ family genes may act as regulators of hematopoiesis under the control of hemopoietic cytokines. |
| isotig16533 | *skNac* |  | ORF |  | insertions | 280 | Gene involved in cardiac and skeletal muscle development [12] |
| isotig40296 | *Eif3a* |  | ORF |  | insertion | 90 | Translation factor essential in development, and involved in cell cycle progression [13] |
| isotig04552 | *Rpl4* |  | ORF |  | insertion | 96 | Protein component of the 60S subunit of the ribosomes. Its cellular distribution is changed during hypoxia [14] leading to changes in translation. |
| isotig42961 | *Serping1* |  | ORF |  | insertion | 54 | *Serping1* gene may be associated to age-related macular degeneration [15] |
| contig64366 | *titin* | Poly PE | √ ORF | × | insertion | 54 | The *S. galili* homologs include short, weakly conserved amino acid repeats in *Spalax*. |
| isotig10050 | *Dicer1* | Poly E/D | √ ORF | × | insertion | <40 |
| isotig17313 | *Parp9* |  | ORF |  | insertion | 54 | *Parp9* binds monoADP ribose and poly(ADP-ribose), and may regulate transcription [16] |

References

1. Wang GS, Hong CJ, Yen TY, Huang HY, Ou Y, et al. (2004) Transcriptional modification by a CASK-interacting nucleosome assembly protein. Neuron 42(1): 113-128.

2. Hsueh YP (2008) Transcriptional regulation of the Tbr1-CASK-CINAP protein complex in response to neuronal activity. Transcriptional Regulation by Neuronal Activity : 51-71.

3. Zannettino AC, Psaltis PJ, Gronthos S (2008) Home is where the heart is: Via the FROUNT. Cell Stem Cell 2(6): 513-514.

4. Hannigan GE, Coles JG, Dedhar S (2007) Integrin-linked kinase at the heart of cardiac contractility, repair, and disease. Circ Res 100(10): 1408-1414.

5. Jiao Q, Shimura M, Akaike T, Ishikawa Y, Minamisawa S (2008) Sarcalumenin plays a critical role in age-dependent reduction in cardiac function and SERCA2a activity. The FASEB Journal 22(1_MeetingAbstracts): 1163.23.

6. Shimura M, Minamisawa S, Takeshima H, Jiao Q, Bai Y, et al. (2008) Sarcalumenin alleviates stress-induced cardiac dysfunction by improving Ca2+ handling of the sarcoplasmic reticulum. Cardiovasc Res 77(2): 362-370.

7. Preta G, de Klark R, Glas R (2009) A role for nuclear translocation of tripeptidyl-peptidase II in reactive oxygen species-dependent DNA damage responses. Biochem Biophys Res Commun 389(4): 575-579.

8. Das-Bradoo S, Nguyen HD, Wood JL, Ricke RM, Haworth JC, et al. (2010) Defects in DNA ligase I trigger PCNA ubiquitylation at lys 107. Nat Cell Biol 12(1): 74-9; sup pp 1-20.

9. Abbasi R, Ramroth H, Becher H, Dietz A, Schmezer P, et al. (2009) Laryngeal cancer risk associated with smoking and alcohol consumption is modified by genetic polymorphisms in ERCC5, ERCC6 and RAD23B but not by polymorphisms in five other nucleotide excision repair genes. International Journal of Cancer 125(6): 1431-1439.

10. Boyadjiev SA, Fromme JC, Ben J, Chong SS, Nauta C, et al. (2006) Cranio-lenticulo-sutural dysplasia is caused by a SEC23A mutation leading to abnormal endoplasmic-reticulum-to-golgi trafficking. Nat Genet 38(10): 1192-1197.

11. Dauffy J, Mouchiroud G, Bourette RP (2006) The interferon-inducible gene, Ifi204, is transcriptionally activated in response to M-CSF, and its expression favors macrophage differentiation in myeloid progenitor cells. J Leukoc Biol 79(1): 173-183.

12. Li H, Randall WR, Du SJ (2009) skNAC (skeletal naca), a muscle-specific isoform of naca (nascent polypeptide-associated complex alpha), is required for myofibril organization. FASEB J 23(6): 1988-2000.

13. Dong Z, Liu Z, Cui P, Pincheira R, Yang Y, et al. (2009) Role of eIF3a in regulating cell cycle progression. Exp Cell Res 315(11): 1889-1894.

14. Thomas JD, Johannes GJ (2007) Identification of mRNAs that continue to associate with polysomes during hypoxia. RNA 13(7): 1116-1131.

15. Lotery A, Ennis S, Gibson J, Cree A, Harris C, et al. (2009) The role of SERPING1 in age related macular degeneration. Acta Ophthalmol 87.

16. Miwa M, Masutani M (2007) PolyADP-ribosylation and cancer. Cancer Sci 98(10): 1528-1535.
