# Supplementary material for: Transcriptome Sequencing of the Blind Subterranean Mole Rat, Spalax galili: Utility and Potential for the Discovery of Novel Evolutionary Patterns
Source: PLoS One. 2011 Aug 12;6(8):e21227. doi: 10.1371/journal.pone.0021227 (PMC3155515; doi:10.1371/journal.pone.0021227)
Supplement: Table S5 — Known functional roles of genes most significantly up-regulated in muscle/hypoxia vs. muscle/normoxia (C.4 vs. C.3) and brain/hypoxia vs. brain/normoxia (C.2 vs. C.1). The genes shown in this table are taken from the entire list of genes up-regulated in muscle/hypoxia vs. muscle/normoxia (table S1) and brain/hypoxia vs. brain/normoxia (table S2). (DOC) [file pone.0021227.s009.doc]

**Table S5.**

| **Table** | **Gene Name** | **Role** | **Ref.** |
| --- | --- | --- | --- |
| S1 | jumonji domain containing 1C | Implicated in the reactivation of silenced genes, and a key regulator of gene expression. |  |
| S1 | glutamate-ammonia ligase (glutamine synthetase) | The higher availability of glutamic acid in anoxia might cause excitotoxicity leading to cell damage. The elevation of glutamine synthetase may have a role in protecting PC12 cells from the cytotoxic effects of glutamate during hypoxia. | [1] |
| S1 | xin actin-binding repeat containing 1 | Implicated in cardiomyopathies, and cardiac hypertrophy. | [2,3] |
| S1 | syndecan 4 | Cell-adhesion molecules involved in the adhesion of cancer cells to fibronectin. | [4] |
| S1 | Titin | The muscle ankyrin repeat proteins that are hypoxia sensitive bind to titin. | [5] |
|  | ankyrin repeat domain 1 | Upregulated in hypoxia in *Spalax*. | [5] |
| S1 | forkhead box O1 | Involved in anoxia and hypoxia responses. | [6] |
| S1 | activating transcription factor 3 | TF involved in anoxia and hypoxia. | [7] |
| S1 | stomatin; ABO-family member 5 | Expressed in blood cells. |  |
| S1 | zinc finger and BTB domain containing 16 | Involved in blood cell development and differentiation. | [8] |
| S1 | DNA-damage-inducible transcript 4 | HIF-1 target gene. | [9] |
| S1 | zinc finger, AN1-type domain 5 | HIF-1 target gene; osteoclast differentiation. | [10] |
| S1 | connective tissue growth factor | Increased following stroke. | [11] |
| S1 | DIP2 disco-interacting protein 2 homolog A | DIP2A functions as a novel receptor that mediates the cardiovascular protective effects of FSTL1. | [12] |
| S1 | pyruvate dehydrogenase kinase, isozyme 4 | Induced during hypoxia. Inhibits oxidative decarboxylation of pyruvate in the mitochondria. | [13] |
| S1 | lipin 1 | Lipin 1 effects adipose tissue mass, insulin sensitivity and glucose homeostasis. Exhibits a strong interdependence with vascular endothelial growth factor (VEGF) gene expression. | [14] |
| S1 | chemokine receptor 7 | Important for angiogenesis. | [15] |
| S2 | actinin alpha 4 | An adapter protein that may anchor INOS (NO synthase) to the actin cytoskeleton and regulates NO levels. NO might be important for hypoxia signaling and deprivation of the arteries of NO might lead to initiation of delayed vasospasm. | [16,17] |
| S2 | cytochrome c oxidase subunit IV isoform 1 | HIF-1 regulates cytochrome oxidase subunits to optimize efficiency of respiration in hypoxic cells. |  |
| S2 | cytochrome P450, family 4, subfamily b, polypeptide 1 | Cytochrome P450 epoxygenases 2C8 and 2C9 are implicated in hypoxia-induced endothelial cell migration and angiogenesis. | [18] |
| S2 | solute carrier family 23 member 2 | SVCT2 (the product of SLC23A2) possibly regulates HIF-1 alpha. | [19] |
| S2 | myc induced nuclear antigen | Interplay between N-Myc and HIF-1α plays a critical role in neuroblastoma. For example, high levels of N-Myc override HIF-1α inhibition of cell cycle progression enabling continued proliferation under hypoxia. Furthermore, both HIF-1α and N-Myc are essential for the Warburg effect (aerobic glycolysis) in neuroblastomas by activating the transcription of multiple glycolytic genes. | [20] |
| S2 | Phosphofructo-kinase, muscle | A key regulatory enzyme of glycolysis. Phosphofructo-1-kinase deficiency leads to a severe cardiac and hematological disorder | [21] |
| S2 | prostaglandin D2 synthase (brain) | Shear stress induces transcription of prostaglandin D2 synthase (L-PGDS). L-PGDS expression is induced in the myocardium to cope with hemodynamic stress that is generated by hypoxemia. | [22] |
| S2 | 6-phosphofructo-2-kinase | HIF-1 target gene. A key step in controlling glycolytic rate is the conversion of fructose-6-P to fructose-1,6-P(2) by 6-phosphofructo-1-kinase (PFK-1). 4) | [23] |
| S2 | hemoglobin, beta | Oxygen transport in red blood cells. Upregulated in hypoxia and ischemia. | [24] |
| S2 | nephroblastoma overexpressed gene | HIF-1 target gene. | [25] |

References

1. Kobayashi S, Millhorn DE (2001) Hypoxia regulates glutamate metabolism and membrane transport in rat PC12 cells. J Neurochem 76(6): 1935-1948.

2. Otten J, van der Ven PF, Vakeel P, Eulitz S, Kirfel G, et al. (2010) Complete loss of murine xin results in a mild cardiac phenotype with altered distribution of intercalated discs. Cardiovasc Res 85(4): 739-750.

3. Wang Q, Lin JL, Reinking BE, Feng HZ, Chan FC, et al. (2010) Essential roles of an intercalated disc protein, mXinbeta, in postnatal heart growth and survival. Circ Res 106(9): 1468-1478.

4. Koike T, Kimura N, Miyazaki K, Yabuta T, Kumamoto K, et al. (2004) Hypoxia induces adhesion molecules on cancer cells: A missing link between warburg effect and induction of selectin-ligand carbohydrates. Proc Natl Acad Sci U S A 101(21): 8132-8137.

5. Band M, Joel A, Avivi A (2010) The muscle ankyrin repeat proteins are hypoxia-sensitive: In vivo mRNA expression in the hypoxia-tolerant blind subterranean mole rat, *Spalax* *ehrenbergi*. J Mol Evol 70(1): 1-12.

6. Zhan L, Wang T, Li W, Xu ZC, Sun W, et al. (2010) Activation of Akt/FoxO signaling pathway contributes to induction of neuroprotection against transient global cerebral ischemia by hypoxic pre-conditioning in adult rats. J Neurochem 114(3): 897-908.

7. Chen SC, Liu YC, Shyu KG, Wang DL (2008) Acute hypoxia to endothelial cells induces activating transcription factor 3 (ATF3) expression that is mediated via nitric oxide. Atherosclerosis 201(2): 281-288.

8. Dick JE, Doulatov S (2009) The role of PLZF in human myeloid development. Ann N Y Acad Sci 1176: 150-153.

9. Ding M, Xu JY, Fan Y (2010) Altered expression of mRNA for HIF-1alpha and its target genes RTP801 and VEGF in patients with oral lichen planus. Oral Dis 16(3): 299-304.

10. Knowles HJ, Cleton-Jansen AM, Korsching E, Athanasou NA (2010) Hypoxia-inducible factor regulates osteoclast-mediated bone resorption: Role of angiopoietin-like 4. FASEB J 24(12): 4648-4659.

11. Leeuwis JW, Nguyen TQ, Theunissen MG, Peeters W, Goldschmeding R, et al. (2010) Connective tissue growth factor is associated with a stable atherosclerotic plaque phenotype and is involved in plaque stabilization after stroke. Stroke 41(12): 2979-2981.

12. Ouchi N, Asaumi Y, Ohashi K, Higuchi A, Sono-Romanelli S, et al. (2010) DIP2A functions as a FSTL1 receptor. J Biol Chem 285(10): 7127-7134.

13. Semenza GL (2010) Hypoxia-inducible factor 1: Regulator of mitochondrial metabolism and mediator of ischemic preconditioning. Biochim Biophys Acta .

14. Miranda M, Escote X, Ceperuelo-Mallafre V, Megia A, Caubet E, et al. (2010) Relation between human LPIN1, hypoxia and endoplasmic reticulum stress genes in subcutaneous and visceral adipose tissue. Int J Obes (Lond) 34(4): 679-686.

15. Watanabe K, Penfold ME, Matsuda A, Ohyanagi N, Kaneko K, et al. (2010) Pathogenic role of CXCR7 in rheumatoid arthritis. Arthritis Rheum 62(11): 3211-3220.

16. Daniliuc S, Bitterman H, Rahat MA, Kinarty A, Rosenzweig D, et al. (2003) Hypoxia inactivates inducible nitric oxide synthase in mouse macrophages by disrupting its interaction with alpha-actinin 4. J Immunol 171(6): 3225-3232.

17. Hiroi Y, Guo Z, Li Y, Beggs AH, Liao JK (2008) Dynamic regulation of endothelial NOS mediated by competitive interaction with alpha-actinin-4 and calmodulin. FASEB J 22(5): 1450-1457.

18. Michaelis UR, Fisslthaler B, Barbosa-Sicard E, Falck JR, Fleming I, et al. (2005) Cytochrome P450 epoxygenases 2C8 and 2C9 are implicated in hypoxia-induced endothelial cell migration and angiogenesis. J Cell Sci 118(Pt 23): 5489-5498.

19. Harrison FE, May JM (2009) Vitamin C function in the brain: Vital role of the ascorbate transporter SVCT2. Free Radic Biol Med 46(6): 719-730.

20. Qing G, Skuli N, Mayes PA, Pawel B, Martinez D, et al. (2010) Combinatorial regulation of neuroblastoma tumor progression by N-myc and hypoxia inducible factor HIF-1alpha. Cancer Res 70(24): 10351-10361.

21. Garcia M, Pujol A, Ruzo A, Riu E, Ruberte J, et al. (2009) Phosphofructo-1-kinase deficiency leads to a severe cardiac and hematological disorder in addition to skeletal muscle glycogenosis. PLoS Genet 5(8): e1000615.

22. Han F, Takeda K, Ishikawa K, Ono M, Date F, et al. (2009) Induction of lipocalin-type prostaglandin D synthase in mouse heart under hypoxemia. Biochem Biophys Res Commun 385(3): 449-453.

23. Bartrons R, Caro J (2007) Hypoxia, glucose metabolism and the warburg's effect. J Bioenerg Biomembr 39(3): 223-229.

24. He Y, Hua Y, Liu W, Hu H, Keep RF, et al. (2008) Effects of cerebral ischemia on neuronal hemoglobin. Journal of Cerebral Blood Flow & Metabolism 29(3): 596-605.

25. Wolf N, Yang W, Dunk CE, Gashaw I, Lye SJ, et al. (2010) Regulation of the matricellular proteins CYR61 (CCN1) and NOV (CCN3) by hypoxia-inducible factor-1{alpha} and transforming-growth factor-{beta}3 in the human trophoblast. Endocrinology 151(6): 2835-2845.
